# Transcriptomic evidence for visual adaptation during the aquatic to terrestrial metamorphosis in leopard frogs

**DOI:** 10.1186/s12915-022-01341-z

**Published:** 2022-06-28

**Authors:** Ryan K. Schott, Rayna C. Bell, Ellis R. Loew, Kate N. Thomas, David J. Gower, Jeffrey W. Streicher, Matthew K. Fujita

**Affiliations:** 1 Department of Biology, York University, Toronto, Ontario Canada; 2 Department of Vertebrate Zoology, National Museum of Natural History, Smithsonian Institution, Washington DC, USA; 3 Department of Herpetology, California Academy of Sciences, San Francisco, CA USA; 4 Department of Biomedical Sciences, Cornell University College of Veterinary Medicine, Ithaca, NY USA; 5 Department of Life Sciences, The Natural History Museum, London, UK; 6 Department of Biology, Amphibian and Reptile Diversity Research Center, The University of Texas at Arlington, Arlington, TX USA

**Keywords:** Sensory biology, Eye transcriptome, Differential gene expression, Amphibian, Visual plasticity, Microspectrophotometry

## Abstract

**Background:**

Differences in morphology, ecology, and behavior through ontogeny can result in opposing selective pressures at different life stages. Most animals, however, transition through two or more distinct phenotypic phases, which is hypothesized to allow each life stage to adapt more freely to its ecological niche. How this applies to sensory systems, and in particular how sensory systems adapt across life stages at the molecular level, is not well understood. Here, we used whole-eye transcriptomes to investigate differences in gene expression between tadpole and juvenile southern leopard frogs (*Lithobates sphenocephalus*), which rely on vision in aquatic and terrestrial light environments, respectively. Because visual physiology changes with light levels, we also tested the effect of light and dark exposure.

**Results:**

We found 42% of genes were differentially expressed in the eyes of tadpoles versus juveniles and 5% for light/dark exposure. Analyses targeting a curated subset of visual genes revealed significant differential expression of genes that control aspects of visual function and development, including spectral sensitivity and lens composition. Finally, microspectrophotometry of photoreceptors confirmed shifts in spectral sensitivity predicted by the expression results, consistent with adaptation to distinct light environments.

**Conclusions:**

Overall, we identified extensive expression-level differences in the eyes of tadpoles and juveniles related to observed morphological and physiological changes through metamorphosis and corresponding adaptive shifts to improve vision in the distinct aquatic and terrestrial light environments these frogs inhabit during their life cycle. More broadly, these results suggest that decoupling of gene expression can mediate the opposing selection pressures experienced by organisms with complex life cycles that inhabit different environmental conditions throughout ontogeny.

**Supplementary Information:**

The online version contains supplementary material available at 10.1186/s12915-022-01341-z.

## Background

Most animals exhibit complex life cycles with distinct larval and adult life stages (linked by metamorphosis) that occupy different ecological niches and experience distinct selective pressures [1, 2]. Animals with simple life cycles may be phenotypically constrained due to genetic correlations between juvenile and adult morphologies, whereas complex life cycles in which animals transition through two or more distinct phenotypic phases may disrupt these constraints through adaptive decoupling [2]. This hypothesis predicts that each life stage can adapt more freely to its particular ecological niche [2, 3]. Adaptive decoupling has been documented in different taxa, including marine invertebrates, insects, and amphibians, and across many types of traits, including morphology, coloration, immunity, bacterial symbiosis, and social behavior [3–8]. The degree of decoupling can vary considerably, and in many cases, larval trait characteristics heavily influence how those traits manifest in adults [9–14]. Investigating changes in gene expression across life stages can be an important step towards understanding the genetic basis of adaptive decoupling, or alternatively, high genetic correlation [3, 4, 7, 15]. Our study provides important insight into adaptive decoupling in the visual system by quantifying differences in gene expression profiles across life stages in frogs.

The adaptive decoupling hypothesis has been studied in anuran amphibians (frogs and toads) due to their complex life cycles that typically include distinct larval (tadpole) and adult stages linked by metamorphosis. Although both tadpole and adult ecologies vary extensively across the frog tree of life, most species have aquatic and herbivorous tadpoles, while adults are generally more terrestrial and carnivorous [16]. A number of studies have shown that morphological diversity of tadpoles and adult frogs is decoupled [6, 17–19] and that genetic correlations between tadpole and adult traits are low in many, but not all, cases [20–23]. However, several aspects of frog biology do tend to be coupled across life stages to varying degrees including behavior [24], size [14, 25], and developmental plasticity [26]. In general, the degree of decoupling across anuran life stages is trait, and possibly taxon, dependent.

The visual system of frogs is particularly compelling in the context of adaptive decoupling because most species use vision to sense their environments as both tadpoles and adults, but differences in morphology, ecology, and behavior between life stages likely place different selective pressures on the visual system across ontogeny. Correspondingly, intraspecific eye-body size allometry across ontogeny varies widely across anurans with a shift at metamorphosis in several species, suggesting that eye growth is partially decoupled and is shaped by both tadpole and adult visual requirements [27]. Eye position also shifts in many anurans from a lateral position in tadpoles to a more frontal position in adults, resulting in binocular overlap [28]. Other morphological and physiological changes to the visual system that may also occur between larval and adult life stages in anurans include the loss or development of accessory structures (e.g., umbracula, elygia, eyelids, nictitating membranes), changes in photoreceptor and ganglion cell morphology and abundance, and shifts in synaptic connections. As with most changes associated with metamorphosis, changes to the visual system are broadly controlled by thyroid hormone [28, 29]. The specific molecular basis of these broad morphological and physiological changes to the visual system, however, are poorly understood, especially in species that transition from aquatic tadpoles to terrestrial adults (the frog model species, *Xenopus laevis*, is fully aquatic both as a tadpole and as an adult).

Aquatic and terrestrial environments differ in both the intensity and spectral composition of available light, and thus, the visual systems of anurans with aquatic larvae and terrestrial adults can encounter vastly different light environments. Water preferentially absorbs and scatters the shorter (ultraviolet–violet) and longer (yellow–red) wavelengths of light resulting in a narrowing of the spectrum and overall reduction in light availability. In clear water, this results in a depth-dependent blue shift in available light [30]. Freshwater environments often have dissolved organic and particulate matter that absorbs shorter (violet and blue) wavelengths, resulting in a red-shifted light environment, and these particles may also further reduce the penetration of light with depth [31, 32]. Many vertebrate animals that inhabit turbid, red-shifted aquatic environments use visual pigments that have red-shifted sensitivity (relative to species in marine or terrestrial environments) that presumably match the available light more closely [33–35]. This shift can be accomplished through the use of a light-sensitive chromophore derived from vitamin A_2_ (3,4-didehydroretinal) in contrast to “typical” vertebrate visual pigments that contain a vitamin A_1_ derived chromophore (retinal). Consequently, some frog species that transition from an aquatic tadpole to a terrestrial adult have a corresponding shift in chromophore usage from mainly A_2_ to predominantly, or exclusively, A_1_ [34]. By contrast, African clawed frogs, *X. laevis*, are fully aquatic throughout their lifecycle and exclusively use the A_2_ chromophore [36]. The conversion from the A_1_ to A_2_ chromophore is mediated by a cytochrome enzyme encoded by *CYP27C1* [37], and thus differential expression of this gene in aquatic versus terrestrial life stages likely plays an important role in maximizing visual sensitivity in these distinct light environments.

Differential visual opsin usage and expression is another potential mechanism of adaptation to the changing light conditions that tadpoles and adult frogs experience across life stages. Visual opsins are the protein components of visual pigments, and changes to the protein sequence can affect spectral sensitivity. Vertebrates ancestrally have five classes of visual opsins (LWS, RH1, RH2, SWS1, SWS2) that are sensitive to different portions of the visual spectrum, although gene loss and duplication are common in some lineages [38]. Frogs, for instance, have lost RH2 based on evidence from whole genomes and retinal mRNA [39], whereas teleost fishes often have additional, duplicated copies of some opsin classes [40]. Differential visual opsin usage and expression across life stages is employed by many teleosts, particularly when larval habitat or foraging differs from that of adults [40]. For example, Midas cichlids express *SWS1* only early in ontogeny and express *SWS2* only, or primarily, late in ontogeny. Expression of other opsin genes also varies significantly over ontogeny [41]. Whether anurans also use this strategy has yet to be investigated.

Aquatic and terrestrial environments also have different optical properties that apply divergent selective pressures on the lens. In water, the cornea (the outer casing of the eye) has little to no focusing power due to the similar refractive indices of water and the fluid within the eye (aqueous humor [42]). As a result, the lens alone is responsible for focusing images on the retina in aquatic settings. By contrast, in air the cornea has substantial focusing power that varies based on its curvature, and thus a high-powered lens suitable for an aquatic environment would result in over-focusing [42]. In anurans with terrestrial adult life stages, the lens becomes flatter during metamorphosis, reducing its power [28, 43, 44], whereas lens shape changes little in frogs that remain aquatic as adults [45]. The protein composition (crystallins) of the anuran lens may also change during ontogeny, although this appears to occur as lens diameter increases rather than specifically at metamorphosis [46]. For instance, *Lithobates pipiens*, *L. catesbeianus*, and *X. laevis* show a shift from predominantly γ-crystallins to ɑ- and β-crystallins as eye and lens diameter increase [28, 47, 48]. Thus, genes that regulate lens growth and composition may also be differentially expressed through ontogeny, and this has yet to be explored in anurans.

Finally, the plasticity of visual gene expression in larval and adult anurans, as well as other non-model vertebrates, is poorly understood. In particular, the effect of short-term light or dark exposure is one axis of variation that may be important to consider with respect to experimental design in vision research. Animals are often exposed to dark conditions for several hours (i.e., dark adapted) prior to sampling to aid in dissection of the retina and to ensure photoreceptors have not been bleached (activated). The isolated retina is then used for downstream applications such as RNA sequencing or microspectrophotometry (MSP). By contrast, studies that use whole eyes to assess gene expression (e.g., whole-eye transcriptome sequencing) may not use dark adaptation prior to sampling and in general may have more variable sampling conditions, especially when individuals are sampled in the field. Consequently, variability in visual gene expression with light conditions could have important, unappreciated implications for comparative studies of visual evolution.

Here, we used whole-eye transcriptome sequencing of the southern leopard frog (*Lithobates sphenocephalus*) to test for differential expression between fully aquatic tadpoles and post-metamorphic, terrestrial juveniles. This common species is native to freshwater and adjacent habitats throughout the southeastern United States. Tadpoles are fully aquatic and primarily diurnal, grazing on algae and taking shelter in macrophytes and emergent vegetation for the first few months of their lives before undergoing metamorphosis [49, 50]. By contrast, juvenile and adult frogs are terrestrial and nocturnal, spending the day hiding in vegetation, often near water [51]. Although they may jump into water to avoid predators, post-metamorphic frogs (juveniles and adults) are primarily active on land where they feed on terrestrial arthropods and invertebrates [51]. Leveraging the complex life cycle of the southern leopard frog, which use their visual systems in distinct environments and for different tasks at each life stage, we (1) make broad transcriptome-wide comparisons to test for potential adaptive decoupling in gene expression between tadpole and juvenile eyes, and plasticity in response to light exposure, (2) use a curated subset of the genes expressed in the eye that are known to function in the initial stages of vision and in eye and retinal development (visual genes) to specifically test for differential expression between life stages and light treatments, and (3) use MSP to survey spectral absorbance of tadpole and adult photoreceptor cells to bolster the conclusions we draw from the results of our differential expression analyses. This study provides a first look at how molecular aspects of the visual system change in a biphasic vertebrate that transitions from an aquatic to a terrestrial light environment and how variable these changes are with respect to light conditions during sampling.

## Results

### Transcriptome sequencing and assembly

We sequenced whole-eye transcriptomes from six tadpole and six juvenile southern leopard frogs (*Lithobates sphenocephalus*) collected from a wild population in Texas. Three samples from each life stage were exposed to ambient light for 12 h before sampling, while the other three were exposed to complete darkness for 12 h before sampling. Each light treatment group contained one earlier stage tadpole (Gosner stage 25–28) and two from later stages (30–34 in the light treatment and 35–38 in the dark treatment; Additional file 1: Table S1). Sequencing resulted in an average of 33 million paired end reads per sample, which was reduced to 22 million on average after quality control (Additional file 1: Table S1). The reference transcriptome assembled de novo with reads from all 12 samples resulted in 684,947 Trinity transcripts with an N50 of 1065, a 98.9% overall realignment rate, and high completeness, with 88.6% of BUSCO (Benchmarking Universal Single-Copy Orthologs) tetrapod orthologs complete and a further 4.2% of BUSCOs fragmented (Additional file 2). We identified one of the 12 samples as a conspicuous outlier in a principal component analysis (PCA) plot of regularized logarithm (rlog) transformed counts (Additional file 1: Fig. S1). Potential explanations for this outlier include a range of factors that are unrelated to the aims of our study including variation in age, size, or condition among our field-caught samples, as well as contamination and/or RNA degradation during dissection and processing of the tissue. Consequently, the outlier was removed, and we used the remaining 11 samples to produce an updated reference transcriptome with 634,894 Trinity transcripts, an N50 of 1085, the same re-alignment rate as the initial transcriptome (98.9%), and a slightly lower BUSCO completeness with 87.4% BUSCOs complete and a further 4.9% of BUSCOs fragmented (Additional file 2). The transcriptome was reduced to a “best set” of transcripts (reduced transcript set; see Methods and [52]), which resulted in a reduction to 66,165 transcripts with an N50 of 2077 and a slight drop in BUSCO scores (87.1% complete and 4.6% fragmented) but a substantial drop in the number of duplicated BUSCOs (50.1% to 6.9%) indicating a substantial reduction in the redundancy of the transcriptome (Additional file 2).

### Transcriptome-wide analyses reveal substantial differential expression between life stages

**Fig. 1:**
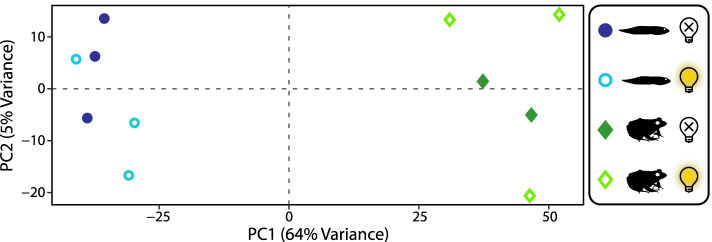
Principal components analysis plot of rlog transformed counts of the whole-eye transcriptome. The first principal component (PC1) accounts for 64% of the variance and clearly separates juveniles and tadpoles. Light and dark exposure are not clearly separated by PC2, which accounts for only 5% of the variance. One of the dark-exposed, juvenile samples was found to be an outlier and was removed prior to this analysis but is shown in Additional file 1: Fig. S1. The PC values are available in Additional file 3

**Fig. 2:**
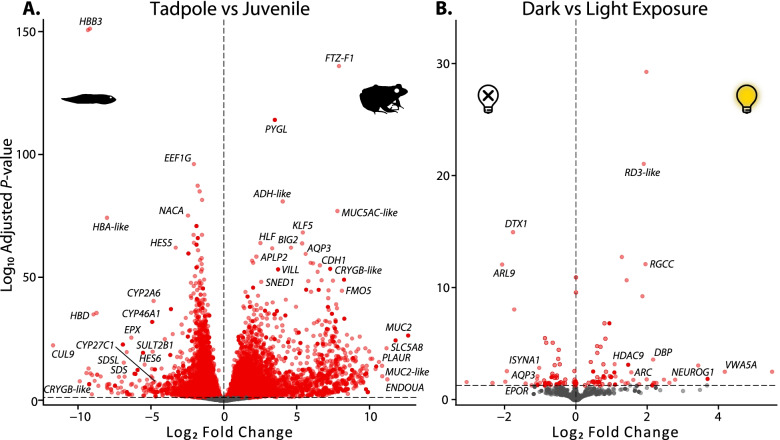
Volcano plots of transcriptome-wide differential expression between **A** tadpoles and juveniles (11,046 transcripts) and **B** dark and light exposure (122 transcripts). Differential expression was estimated with DESeq2 using a multifactor design that accounted for the variation both in the life stages (tadpole vs juvenile) and treatments (dark vs light exposure). For visualization, log_2_ fold changes (LFC) were shrunk and an adjusted *P*-value of 0.05 was set as the significance cut-off. LFC and *P*-values are available in Additional file 3

A PCA of rlog transformed counts showed strong separation of the samples based on life stage (tadpole vs juvenile), but not light vs dark exposure (Fig. 1). Despite our efforts to reduce redundancy, many of the transcripts had zero read counts and thus were not included in the differential expression analysis. Differential expression between tadpoles and juveniles was detected in 11,046 out of 23,019 transcripts (42% significant with an adjusted *P*-value < 0.05) with nonzero total read counts (Fig. 2, Additional file 3). Only 122 transcripts (5% of total) were differentially expressed between light and dark exposure (Fig. 2, Additional file 3). Of the 11,046 transcripts differentially expressed between tadpoles and juveniles, 9038 were annotated with a total of 6721 GO (gene ontology) terms (Additional file 4 [52]). No GO annotations could be found for the remaining 2008 transcripts. Twenty-four GO terms were enriched (*P* < 0.01) with the top three terms being translation, retinol metabolic process, and regulation of small GTPase-mediated signal transduction (Additional file 1: Fig. S2, Additional file 4). While retinol metabolic process, retinoic acid metabolic process, and regulation of synapse structure or activity have implications for visual system development, most other significant terms are likely related to general physiological differences between tadpoles and adults (e.g., oxygen transport, carbohydrate metabolic process, cell morphogenesis). Three other terms related to visual system development were significant at the 0.05 level (positive regulation of neural retina development, *P* = 0.026; photoreceptor cell morphogenesis, *P* = 0.044; retinal pigment epithelium development, *P* = 0.050). GO terms related directly to visual function were not significant, but these were annotated with relatively few terms. For example, only 71 transcripts were annotated as visual perception and 11 as phototransduction (Additional file 4). This is likely the result of relying on a de novo assembly and *X. laevis* for annotation, and so we focused on a curated subset of visual genes for the remainder of the analyses (see below).

### Visual genes are significantly differentially expressed between life stages and light treatments

**Fig. 3:**
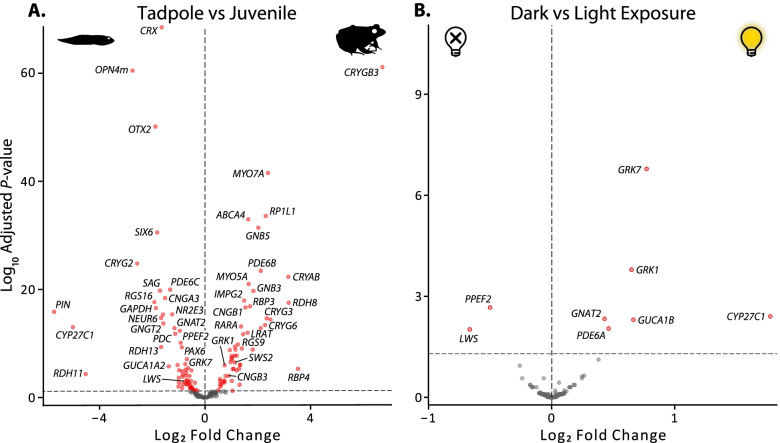
Volcano plots of differential expression of visual gene coding sequences between **A** tadpoles and juveniles (111 genes) and **B** dark and light exposure (8 genes). Differential expression was estimated with DESeq2 using a multifactor design that accounted for the variation in both life stage (tadpole vs juvenile) and treatment (dark vs light exposure). For visualization, log_2_ fold changes (LFC) were shrunk and an adjusted *P*-value of 0.05 was set as the significance cut-off. LFC and *P*-values are available in Additional file 3

To analyze variation within the eye that is specific to the visual system, we generated a second differential expression dataset using a curated subset of 170 visual gene coding sequences that are primarily for genes involved in the initial stages of vision, as well as eye and retinal development. The genes included in the dataset affect numerous aspects of visual function including light detection, lens crystallins, and phototransduction but are not intended to be an exhaustive list all of visual genes expressed in the eye (for details, see the “Methods” section, Additional file 5 [52]). A PCA of rlog transformed counts showed strong separation of the samples based on life stage (tadpole vs juvenile), but not light vs dark exposure, similar to the transcriptome-wide results (Additional file 1: Fig. S3). We found 111 genes (69%) that were identified as being differentially expressed between life stages with an adjusted *P*-value < 0.05 (Fig. 3, Additional file 6). This percentage of significant differential expression is higher than that found transcriptome-wide and may be so, in part, because only functionally relevant visual genes were included in the curated dataset. Several types of visual genes had strong support for differential expression between tadpoles and juveniles, including chromophore usage, visual opsin, phototransduction, and lens crystallin genes that have clear consequences for vision in different light environments and are addressed in more detail below. In addition, tadpoles and juveniles differed in expression of visual and photoreceptor development genes, several non-visual opsins, and a number of visual cycle genes.

Eight genes (5%) were differentially expressed between light and dark-exposed individuals at an adjusted *P*-value of < 0.05 (Fig. 3, Additional file 6), which is the same percentage of differentially expressed genes between light treatments in the transcriptome-wide analysis. The differences observed between the light and dark treatments are unlikely to be due to differences in the developmental stage of the tadpoles because each treatment group contained one earlier stage and two later stage individuals (Additional file 1: Table S1). The differentially expressed genes were a subset of those found between tadpoles and adults, including several phototransduction genes, one visual opsin gene (*LWS*), and a gene involved in chromophore usage (*CYP27C1*, see below).

### *CYP27C1* is upregulated in tadpoles coincident with a red shift of the spectral absorbance of visual pigments

**Fig. 4:**
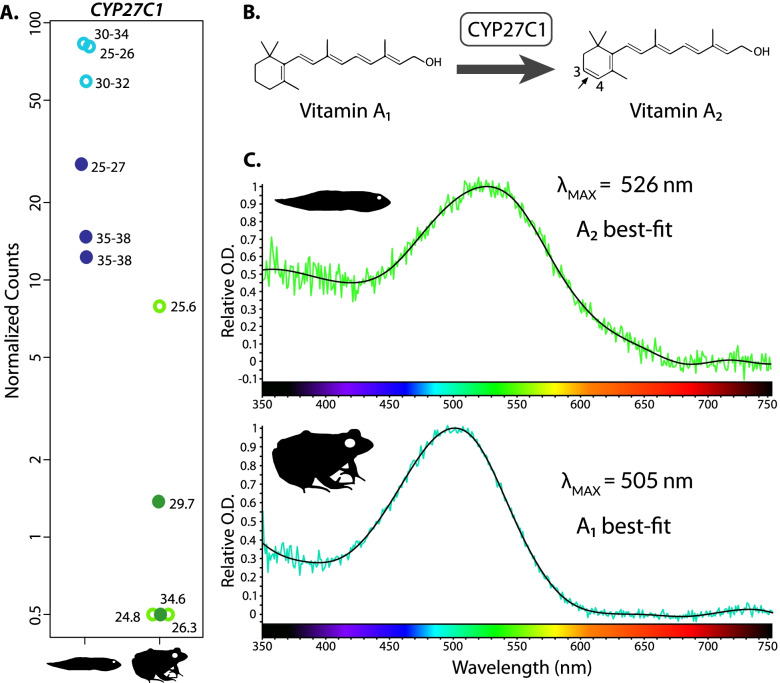
Upregulation of *CYP27C1* in tadpoles corresponds to red-shifted absorbance spectra of visual pigments and use of primarily A_2_ chromophore as detected by microspectrophotometry (MSP). **A** Plots of normalized read counts of *CYP27C1* for light (open) and dark (closed) exposed juveniles and tadpoles. Data are plotted with an additional pseudocount of 0.5 to allow for log plotting. Numbers beside the data points indicate the Gosner stage or size (SVL, mm) of the respective tadpole or juvenile. **B** Conversion of vitamin A_1_ chromophore to vitamin A_2_ chromophore by *CYP27C1*. Based on Enright et al. [37]. **C** Representative MSP absorbance spectra from RH1 (red) rod photoreceptors of an adult and a tadpole. Absorbance spectra were found to be wider and best-fit by the A_2_ Govardovskii et al. [55] template in the tadpole, and narrower and best-fit by the A_1_ Govardovskii template in the adult. Each absorbance spectra curve is coloured to match the wavelength of light at the corresponding λ_max_. Normalized count data for each gene are found in Additional file 6. Absorbance spectra for additional photoreceptor cell types and complete results tables can be found in Additional file 1: Figs. S4–S6 and Table S2, and Additional file 7

We found that *CYP27C1*, which encodes a cytochrome P450 family enzyme that converts A_1_ chromophore into A_2_ in vertebrates [37], was significantly upregulated in tadpoles compared to juveniles and in light-exposed individuals (primarily tadpoles) compared to dark-exposed (Fig. 4). Correspondingly, absorbance spectra of individual RH1 rod photoreceptor cells measured with MSP were red shifted in the tadpole (*n* = 38) relative to the adult (*n* = 18) and fit A_2_ absorbance spectra templates better than A_1_ templates (Fig. 4; Additional file 1: Table S3). Additionally, the absorbance maxima (λ_max_) of tadpole photoreceptors matched values expected from primarily A_2_-based visual pigments (e.g., 526 nm for RH1 rods), while λ_max_ values for adult photoreceptors matched expectations for primarily A_1_-based pigments (e.g., 505 nm for RH1 rods; Fig. 4; Additional file 1: Fig. S4) when compared to previous MSP studies in a closely related leopard frog species (see the “Discussion” section [53, 54]). While not our primary focus, we did estimate λ_max_ and chromophore type for the other photoreceptor types, but this was difficult due to the limited number of highly noisy scans we were able to obtain, especially for the tadpole, due in part to its small size (Additional file 1: Fig. S5; Additional file 1: Table S2). However, the LWS cones appear to follow a similar pattern with a shift from a primarily A_2_-based pigment in tadpoles (λ_max_ of ~ 626 nm) to a primarily A_1_-based pigment in adults (~ 579 or ~ 603 nm; see below and the “Discussion” section for more details).

We also found interesting variation in *CYP27C1* expression patterns among the individual samples. The two tadpoles in later developmental stages (Gosner stage 35–38) had the lowest *CYP27C1* expression, substantially lower than the other dark-exposed tadpole (stage 25–27). By contrast, the three light-exposed tadpoles all had higher expression levels that were much more closely clustered together despite ranging from stages 25–26 to 30–34. This suggests that *CYP27C1* expression may begin to decrease more strongly around stage 35–38, but this could also be due to individual or other sources of variation. In juveniles, we also saw variation in *CYP27C1* expression, with three of the individuals having no expression and two individuals with moderate expression. This variation does not appear to be related to size because both the smallest and largest individuals lacked expression. Of the two juveniles with non-zero expression, the light-exposed individual had substantially higher expression than the dark-exposed individual, consistent with the results for tadpoles.

### Three photoreceptor classes identified by MSP in tadpoles and adults

Based on current evidence, frogs can have two distinct types of rods; two, or possibly three, types of single cones; and, potentially, multiple types of double cones (for a review see [39, 56]). The rods have historically been called “red” and “green” rods but contain a green-absorbing RH1 rod visual pigment and a blue-absorbing SWS2 cone visual pigment, respectively [56]. To avoid confusion, we refer to these, and the other photoreceptor classes, based on their inferred visual pigment (e.g., RH1 rod and SWS2 rod for “red” and “green” rods, respectively). In addition to the two rods, there can be LWS and SWS1 single cones [57–60]. Other cone types have also been identified, but there is only indirect evidence as to the visual pigment they contain. This includes a third type of single cone identified immunocytochemically in *X. laevis* retina [59] and a single cone type with a λ_max_ of 466 nm identified microspectroscopically in *Oophaga pumilio* [61], both of which may contain SWS2 visual pigment. In addition, multiple types of double cone have been identified in frogs, all of which have a presumed LWS primary member with a λ_max_ near 570 nm (for A_1_-based pigments) [53, 54, 62]. In ranids, the accessory member typically contains a ~502 nm pigment indistinguishable from the RH1 rod pigment suggesting an LWS-RH1 pair [53, 54, 62]. One instance of a blue-absorbing 433 nm pigment spectroscopically similar to the SWS2 pigment in “green” rods was observed in an accessory member in *L. catesbeianus* [62]*.* This suggests the possibility of an LWS-SWS2 pair, but this could also be an LWS-SWS1 pair or be the result of coincidental placement of two single cones and not a true double cone [62]. In *X. laevis*, the double cones appear to both contain LWS visual pigment based indirectly on immunocytochemical results [59].

In the southern leopard frog, we identified three photoreceptor classes in both a tadpole and an adult using MSP: SWS2 rods, RH1 rods, and LWS cones (Additional file 1: Figs. S5–S7 and Table S2 and Additional file 7 [52]). We did not find evidence of cones that could contain SWS1 or SWS2, although they may be present, and we did not attempt to evaluate putative double cones. In the tadpole, we obtained only a single measurement of an SWS2 rod and found the absorbance spectra was best fit by an A_1_ chromophore template with a λ_max_ of 433 nm (431 nm when fit with A_2_; Additional file 1: Table S2). Because this was based on one noisy scan, we consider these results to be approximate. In the adult, we found SWS2 rods had an A_1_ best fit with a mean λ_max_ of 437 nm. The tadpole RH1 rods had an A_2_ best fit with a mean λ_max_ of 526 nm, whereas the adult RH1 rods had an A_1_ best fit with a mean λ_max_ of 505 nm. A smaller subset of adult RH1 rods (*n* = 4) had distinct shapes of the absorbance spectra compared to the primary group of RH1 rods (*n* = 18), and these had an A_2_ best fit with a mean λ_max_ of 501 nm (Additional file 1: Fig. S6). However, the mean λ_max_ of this smaller population of RH1 rods was the same as the primary population when they were fit with the A_1_ template (505 nm; Additional file 1: Table S2). As a result, it is unclear whether these actually represent two distinct populations of RH1 rods (but see the “Discussion” section). The tadpole LWS cones had an A_1_ best fit and mean λ_max_ of 635 nm (*n* = 2), but this λ_max_ is inconsistent with a primarily A_1_-based pigment (see [63]), and instead we favour the A_2_-fit mean λ_max_ of 626 nm. In the adult, two distinct groups of LWS cones were identified: one with an A_1_ best fit and a mean λ_max_ of 579 nm (*n* = 2) and a single photoreceptor, also with an A_1_ best fit, but a λ_max_ of 603 nm (Additional file 1: Fig. S5 and Table S2).

### Visual opsin expression varies between life stages and light treatments

**Fig. 5:**
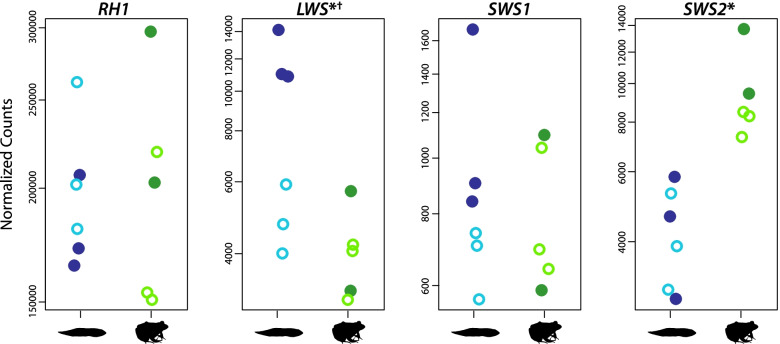
Expression profiles of visual opsin genes. *LWS* and *SWS2* were significantly differentially expressed between juveniles and tadpoles (indicated by the *), although the difference for *LWS* was driven by significant upregulation with dark exposure (indicated by the ^†^), specifically in tadpoles (Additional file 6). Plots show normalized read counts (with an additional pseudocount of 0.5 to allow for log scale plotting) for each gene. Dark and light exposure are denoted by the closed and open circles, respectively. RH1, LWS, and SWS2 photoreceptor cell types were detected by MSP, whereas SWS1 was not (corresponding MSP results in Additional file 1: Figs. S4–S6 and Table S2). Normalized count data for each gene are found in Additional file 6

Each of the four visual opsin genes expected in frogs (*RH1*, *LWS*, *SWS1*, and *SWS2*) was expressed in both tadpoles and juveniles, and we did not detect any evidence of gene duplication or allelic variation. The rod opsin *RH1* was the most highly expressed of the four, likely reflecting the high number of RH1 rod photoreceptor cells typically found in frog retinas. Although our MSP approach is not appropriate for quantitative estimates of photoreceptor abundances, RH1 rod photoreceptors were the dominant cell type we observed and measured (Additional file 1: Table S2), which matches expectations based on studies in *L. pipiens* [53, 64]. *RH1* expression showed high individual variability but did not differ with respect to life stage or light exposure (Fig. 5). The cone photoreceptor opsin genes *LWS* and *SWS2* had similar relative expression levels, and we detected corresponding photoreceptor types for both opsins with MSP (red-sensitive cones and blue-sensitive rods, respectively; Additional file 1: Fig. S5 and Table S2). *SWS2* was significantly upregulated in juveniles compared to tadpoles, while *LWS* was significantly upregulated with dark vs light exposure, although this was driven by high relative expression in tadpoles, specifically. Finally, the cone opsin gene *SWS1* showed the lowest relative expression and, though consistently expressed with some individual variation, showed no shifts associated with life stage or light exposure. Despite the expression of this gene, no short-wavelength-sensitive cones were detected with MSP (Additional file 7).

### Cone phototransduction genes upregulated in tadpoles and rod phototransduction genes upregulated in juveniles

Of the 34 phototransduction genes (excluding visual opsins) that we analyzed, we found 27 to be differentially expressed between tadpoles and juveniles (Additional file 6). Seven of these genes are typically expressed in both rod and cone photoreceptors, while 10 are typically found in cones and 10 typically in rods [65]. Eight of the cone genes were upregulated in tadpoles with two upregulated in juveniles, whereas in rod genes three were upregulated in tadpoles and seven upregulated in juveniles (Additional file 1: Fig. S7, Additional file 6). The difference in the proportion of upregulated cone and rod genes between tadpoles and juveniles was significant (Z-pooled exact test statistic = 2.247, two-tailed *P* = 0.041).

### Expression profiles of lens crystallin genes shift between tadpoles and juveniles

**Fig. 6:**
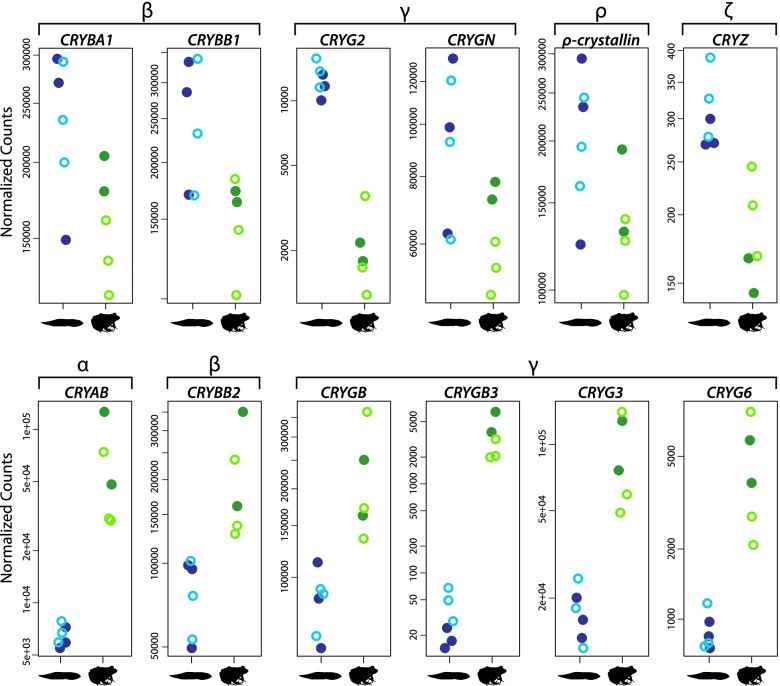
Expression profiles of lens crystallin genes differ substantially between tadpoles and juveniles. Each of the crystallin genes depicted was significantly differentially expressed between juveniles and tadpoles (adjusted *P*-value < 0.05; Additional file 1: Table S3). The top row contains crystallins upregulated in tadpoles, which includes two β-crystallins (*CRYBA1*, *CRYBB1*), two γ-crystallins (*CRYG2*, *CRYGN*), and two taxon-specific crystallins (ρ-crystallin and ζ-crystallin, *CRYZ*). The bottom row contains those upregulated in juveniles, which includes one α-crystallin (*CRYAB*), one β-crystallin (*CRYBB2*), and four γ-crystallins (*CRYGB*, *CRYGB3*, *CRYG3*, *CRYG6*). Plots show normalized read counts (with an additional pseudocount of 0.5 to allow for log scale plotting) for each gene with light and dark exposed samples denoted by the open and closed circles, respectively. Normalized count data for each gene are found in Additional file 6

We analyzed gene expression patterns for 21 ubiquitous crystallins (two ɑ-crystallins, six β-crystallins, and 13 γ-crystallins) [66] and two “taxon-specific” crystallins previously reported in frogs (ρ-crystallin and ζ-crystallin) [67, 68]. Of these, two β-crystallin (*CRYBA1*, *CRYBB1*), two γ-crystallin (*CRYG2*, *CRYGN*), and both taxon-specific crystallin genes were significantly upregulated in tadpoles (Fig. 6, Additional file 5). One ɑ-crystallin (*CRYAB*), two β-crystallin, and four γ-crystallin genes were significantly upregulated in juveniles. Overall changes in relative expression of ɑ-, β-, and γ-crystallin genes were investigated using TMM cross-normalized expression values. Relative expression of γ-crystallin genes was more than twice that of ɑ- and β-crystallin genes in tadpoles (691,387 vs 296,175; Additional file 1: Fig. S8 and Table S3) and slightly less than twice that in juveniles (591,814 vs 313,789). Relative expression of ɑ-crystallin genes was higher in juveniles (96,040 vs 132,927), while β-crystallin gene expression was higher in tadpoles (200,135 vs 180,862). Compared to the ubiquitous crystallins, the two taxon-specific crystallin genes made up a relatively small proportion of the crystallin expression (3.8%) with ρ-crystallin comprising 99.9% of this expression. The relatively low expression of ζ-crystallin suggests it may not be a component of the lens in leopard frogs and may instead be present at housekeeping levels within the eye, because this protein has additional roles outside of the lens, at least in mammals (see [69] and references therein). Finally, we analyzed expression patterns of five other taxon-specific crystallins that have not specifically been identified in frog lenses in previous studies, and one (α-enolase/ENO1/τ-crystallin) with mixed evidence [70, 71]. Of these, three were differentially expressed (two upregulated in tadpoles, one in juveniles), including α-enolase (Additional file 1: Fig. S9). We found no evidence for an effect of light exposure on the expression of any of the lens crystallin genes.

To explore potential functional consequences of changes to lens crystallin composition, we estimated protein refractive index increments (*dn*/*dc*) for the ubiquitous and known frog taxon-specific crystallins (Additional file 1: Fig. S10 and Table S3, Additional file 8). The *dn*/*dc* value defines how much a particular protein will contribute to the refractive index at a given concentration [72]. Of the ubiquitous crystallins, leopard frog ɑ-crystallins were estimated to have the lowest *dn*/*dc* values (0.1941), followed by β-crystallins (0.1970), and γ-crystallins (0.2021). The ɑ-crystallin that was significantly upregulated in juveniles (encoded by *CRYAB*) had the lowest *dn*/*dc* of any of the ubiquitous crystallins (0.1934). The two β-crystallins encoded by significantly upregulated genes in tadpoles had higher *dn*/*dc* values (CRYBA1, 0.1983; CRYBB1, 0.1952) than the β-crystallin upregulated in juveniles (0.1948). The pattern among the differentially expressed γ-crystallins was less clear. The two upregulated γ-crystallins in tadpoles both have relatively high *dn*/*dc* values, but the four upregulated in juveniles had a mix of values. The γ-crystallins, in particular, also had highly variable expression levels and therefore would likely contribute very different amounts to the overall refractive index. When accounting for expression levels (TMM) of all the ubiquitous crystallin genes, tadpoles and juveniles had similar weighted mean *dn*/*dc* values (0.2010 and 0.2003, respectively; Additional file 8). The frog-specific crystallins had lower estimated *dn*/*dc* values than any of the ubiquitous crystallins (ρ-crystallin, 0.1916 and ζ-crystallin, 0.1884; Additional file 1: Table S3).

## Discussion

### Significant shifts in gene expression across ontogeny

The adaptive decoupling hypothesis suggests that animals that develop through two or more distinct phenotypic phases may be able to disrupt genetic correlations between phases, allowing each phase to better adapt to its particular selective environment [3]. This strategy would be particularly relevant for frogs, which often have larval (tadpole) and adult phases that occupy different ecological niches. Although previous studies have shown that morphological diversity of tadpoles and adult frogs is decoupled [6, 17–19], studies testing for differences in gene expression are more limited [18]. We found that a large proportion of the eye transcriptome (42%) was significantly differentially expressed between tadpole and juvenile southern leopard frogs, suggesting substantial, and potentially adaptive, decoupling at the level of gene expression. Further, this suggests that cellular composition, density, and size, in addition to within-cell changes in expression, differ between tadpoles and juvenile frogs. These results are consistent with a recent study of eye-body size allometry across ontogeny in anurans that detected a shift at metamorphosis in several species [27], suggesting that relative eye growth is partially decoupled as well. The extent of decoupling in gene expression we detected was substantially more than was found in a comparison of whole body (excluding gut) transcriptomes of tadpole and adult *Mantidactylus betsileanus* Madagascar frogs, which found that only 14% of annotated transcripts were differentially expressed [18]. In that whole body comparison, differentially expressed transcripts were significantly enriched for genes involved in morphological development, suggesting that phenotypic evolution across phases was decoupled. This observation is consistent with divergent macroevolutionary patterns of body shape diversification in tadpoles versus adult frogs [6]. A comparison of whole-body and whole-eye transcriptomes in both *M. betsileanus and L. sphenocephalus* would clarify whether the observed differences in the degree of differential expression are species or tissue specific.

Other examples of decoupling in gene expression come from insects [4, 73–75]. For instance, in the hymenopteran *Neodiprion lecontei*, which has multiple life stages separated by increasingly dramatic metamorphic transitions, Herrig, et al. [75] found that a progressively greater proportion of genes (up to 31%) were differentially expressed between each successive pair of stages. That observation matched the authors’ predictions that gene expression decoupling would be strongest between the most ecologically dissimilar life stages. Consequently, we propose that expression may be more strongly coupled in frog species that remain aquatic after metamorphosis or in those that do not have a tadpole life stage and instead complete development within the egg and hatch as juvenile frogs (direct development). Studies comparing species that differ in life history and with finer scale ontogenetic sampling are needed to test this hypothesis. Furthermore, studies of immune system activity in *Drosophila* found support for both coupled and decoupled expression of different immune genes [4], underscoring the need for more detailed studies even within broad functional classes of genes.

Although our results have interesting implications for the broad decoupling of gene expression between tadpoles and juveniles, our focus was on visual genes. We found that the visual system was disproportionately decoupled compared to the full eye transcriptome, with significant differential expression between tadpole and juvenile frogs in over half of the visual genes we analyzed. This suggests the visual system may be particularly strongly adapted, through decoupling, to the larval and adult environments. Teleost fishes also show differences in visual gene expression across ontogeny, specifically with regard to visual opsin expression, when larval habitat or foraging differs from that of adults [40]. Changes in expression patterns of other visual genes in teleosts remain to be evaluated, but we expect considerable variation in those taxa with distinct larval and adult ecologies. Likewise, it has recently been proposed that the visual system of the tuatara (*Sphenodon punctatus*) may be uniquely adapted to accommodate differing juvenile and adult ecologies due to the constraints imposed by a single life stage [76]. Tuatara juveniles often take up a diurnal and arboreal lifestyle to avoid the terrestrial, nocturnal, adults that may predate them, and this appears to have resulted in a visual system with a unique mix of diurnal and nocturnal features and highly conserved visual genes compared to other vertebrates [76]. Consequently, gene expression decoupling across ontogeny may also feature in tuatara despite the absence of a complex life cycle. Overall, these studies suggest that differential expression of visual genes may be a fairly widespread strategy in vertebrates that adapt to different light environments across ontogeny.

The visual genes we identified as differentially expressed between tadpole and juvenile leopard frogs affect a broad range of visual functions, including developmental genes, such as *CRX* and *OTX2*, that are important for the differentiation and maintenance of rod and cone photoreceptor cells [77]. Several non-visual opsins were also differentially expressed, including melanopsin (*OPN4m*) and pinopsin (*PIN*), suggesting that circadian clocks and regulation may differ between tadpoles and juveniles [78]. A number of visual cycle genes were differentially expressed, which is likely to have further enabled the partially independent adaptation of the leopard frog visual system to the larval and adult environments. Some of these genes were also differentially expressed between the light- and dark-exposure treatments, which may reflect diel expression patterns or an ability to plastically acclimate to different light environments at the level of gene expression. Finally, the chromophore usage, visual opsin, phototransduction, and lens crystallin genes also had strong support for differential expression between tadpoles and juveniles and have clear consequences for vision in different light environments, which are discussed in more detail below.

### Significant shifts in gene expression with light exposure

In addition to differential gene expression across ontogeny, we also found a smaller set of genes (5% of total) were differentially expressed between dark and light treatments, which was consistent across the full transcriptome and the visual gene datasets. This experimental design was primarily motivated by ongoing comparative studies by our group, and others, that use eye and retinal transcriptomes for molecular evolutionary analyses. In many cases, precise control of lighting environments is not possible (e.g., field collected samples), and we aimed to explore the potential impacts of light exposure variation on visual gene recovery from transcriptomes. Our results suggest that variation in light conditions during sampling is unlikely to have much impact on comparative studies that aim to recover visual genes from whole-eye transcriptomes and conduct molecular evolutionary analyses. However, we did find that several phototransduction genes (e.g., rod and cone opsin kinase [*GRK1* and *GRK7*], cone transducin [*GNAT2*]), one visual opsin (*LWS*), and the chromophore usage gene (*CYP27C1*) were significantly differentially expressed between light and dark exposure. Consequently, we infer that controlled light environments and sampling conditions are likely necessary for accurately assessing differential expression of visual genes in comparative studies. In addition, we suggest that variation in gene expression between light and dark treatments may represent a form of adaptive plasticity to short-term changes in light levels that warrants further investigation both within frogs and in other vertebrates.

### Chromophore usage and light sensitivity shift across ontogeny

Changes in visual pigment light sensitivity associated with the use of the A_1_ and/or A_2_ chromophores have been established using spectrophotometry of retinal extracts and MSP of intact photoreceptors in several tadpole and adult frogs (reviewed in [34]). Recently, the protein responsible for converting A_1_ into A_2_, CYP27C1, was identified ([37]; for a review, see [79]). We found that *CYP27C1* was significantly upregulated in southern leopard frog tadpoles compared to juveniles and, with MSP, confirmed that this difference is associated with red-shifted absorbance spectra and A_2_ chromophore usage in tadpole RH1 rods. We inferred shifts in chromophore usage based on best-fit absorbance templates for RH1 rods in each life stage (A_2_ in tadpoles vs A_1_ in adults) and on the agreement of the λ_max_ values (primarily A_2_-based pigments in tadpoles and A_1_-based pigments in juveniles) with the closely related species *Lithobates pipiens* (see below). Thus, our differential expression and MSP results provide evidence for the role of *CYP27C1* in switching chromophore usage and, correspondingly, spectral sensitivity during ontogeny. Several species of frog that transition from aquatic tadpoles to terrestrial or semi-terrestrial adults, including the tree frogs *Hyla regilla* and *H. arborea*, several other *Lithobates* species (*L. catesbeianus*, *L. clamitans*, *L. esculenta*, *L. pipiens*, *L. sphenocephalus*), *Rana* species (*R. temporaria*, *R. arvalis*), European fire-bellied toad (*Bombina bombina*), and common spadefoot (*Pelobates fuscus*) shift their chromophore usage from primarily A_2_ to primarily A_1_ at metamorphosis [34, 53, 80–82]. The timing of the shift between chromophores in rods varies, with some species (*H. arborea* and *B. bombina*) showing a continuous change from A_2_ to A_1_ during tadpole development, while in other species (*P. fuscus* and *R. arvalis*), the shift does not occur until the appearance of forelimbs [82]. Our data suggest the possibility that the transition may begin at Gosner stage 35–38 in leopard frogs based on the relatively lower *CYP27C1* expression compared to the earlier stages (25–34), even accounting for dark exposure. More data will be needed, however, to further evaluate this timing. In *H. arborea*, the transition from A_2_ to A_1_ was complete post-metamorphosis, with juveniles and adults having the same spectral sensitivities [82]. Our data suggest some juvenile leopard frogs maintain a small proportion of A_2_ pigment based on continued expression of *CYP27C1*, while other specimens showed no expression and thus would be expected to have no A_2_ pigment. By contrast, the toads *Bufo boreas* and *B. bufo* appear to use only A_1_ as both aquatic tadpoles and terrestrial adults [80, 83], and *X. laevis*, which remain fully aquatic as adults, use exclusively A_2_ pigments at both life stages [36].

The mechanism that controls ontogenetic changes in *CYP27C1* expression, and thus, the switch from A_2_ to A_1_ chromophore (or vice versa) is not known but appears to involve thyroid hormone signaling. In most teleost fishes that have been studied, the application of thyroid hormone results in an increase in A_2_ chromophore, but in bullfrog, it results in a reduction in A_2_ [79]. In frogs, application of thyroid hormone can trigger or accelerate metamorphosis, which in turn results in the shift from A_2_ to A_1_ in some species but not others [34] raising the question of how this difference among species is controlled. Additional studies quantifying ontogenetic variation in chromophore usage and *CYP27C1* expression across a greater diversity of frogs may provide a better understanding of the underlying mechanisms and how this trait is associated with changes in visual ecology across species.

Light exposure can also affect the proportion of A_1_ to A_2_ chromophore in the eye. This pattern has been demonstrated as a reversible change in tadpoles of several species of *Lithobates*, where exposure to darkness over extended periods (weeks to over a month) decreased the proportion of A_2_ chromophore, which could be reversed to near normal levels in as little as 24–48 h of constant illumination [81, 84]. We found that *CYP27C1* expression was significantly reduced in dark-exposed compared to light-exposed tadpoles (12 h of exposure), which suggests *CYP27C1* expression is light-dependent in tadpoles and that reduced *CYP27C1* expression upon dark exposure results in a lower proportion of A_2_-based visual pigments. A potential mechanism for light-dependent expression of *CYP27C1* is unknown, but previous experiments suggest this response can be localized to a single eye. Specifically, Bridges [81] found that exposing tadpoles to light after a period of darkness resulted in an increase in A_2_ in unobstructed eyes, but not in eyes that were covered and thus unexposed to light. The functional benefit of this change may be related to the lower dark noise (thermal activations in the absence of photon absorbance) of A_1_ pigments compared to A_2_ pigments, resulting in higher light sensitivity of A_1_ pigments [85]. Thus, exposure to dark conditions could result in increased overall light sensitivity of the tadpole through reduced expression of *CYP27C1*, which in turn reduces the production of A_2_ chromophore and, correspondingly, increases the proportion of the higher-sensitivity A_1_ pigments. This interpretation is perhaps confounded by the observation that some teleost fishes have the opposite reaction to dark exposure, where the proportion of A_2_ pigment increases [34], although at least one teleost species has the same response as tadpoles [86]. This variation suggests there may be other factors beyond spectral tuning and light sensitivity that influence chromophore usage.

### Visual opsins and phototransduction genes are differentially expressed between life stages

Differential expression of visual opsins and phototransduction genes is a second approach that organisms can employ to adapt the sensitivity of their visual system to changes in the light environment. Studies investigating the evolutionary and ecological context of this particular strategy in vertebrates have largely been restricted to the visual opsins of teleost fishes, which tend to have many more (duplicated) copies of these genes than do tetrapods [40]. Some teleost species express only a subset of their total visual opsin repertoire during a particular life stage, which is likely beneficial when larval habitat and associated traits, such as foraging behavior, differ from those of adults [40]. Most frog species make even more dramatic transitions in habitat and foraging behavior across ontogeny, but with far fewer opsins to choose from, their ability to use distinct opsins for larval and adult vision is likely more constrained. Despite this, we found differential expression between tadpoles and adults in two of their four visual opsin genes (*SWS2* and *LWS*). In frogs and salamanders, *SWS2* is expressed in blue-absorbing “green” rods (SWS2 rods), a unique type of rod not found in other vertebrates that, at least in some species, enables color vision at scotopic light levels when cones are inactive [87]. The increased expression of *SWS2* in juvenile leopard frogs suggests an increased proportion of SWS2 rods in the retinas of juveniles relative to tadpoles, which may reflect increased reliance on nocturnal color vision in terrestrial life stages. The significant difference in *LWS* expression, however, appears to be driven by the effect of dark exposure in tadpoles, reflecting differences in expression in the same cell population due to environmental variation. Previous studies in teleost fishes have found variable responses of opsin expression in different light conditions [40, 88] including expression changes with respect to housing animals in different light environments (e.g., clear vs tea-stained water [89, 90]) and daily (diel) variation in expression [91, 92]. One common observation in daily expression cycles is that cone opsin expression peaks near the onset of darkness and remains high throughout the night [92, 93]. Such variation has not yet been characterized in anurans, and future studies with broader taxonomic sampling, and a greater range of light/dark treatments, would improve our understanding of visual gene expression plasticity in larval and adult anurans.

Although we found *SWS1* opsin gene expression in both tadpole and adult life stages, we did not find evidence for any short-wavelength-sensitive (SWS) cone photoreceptors that could be attributed to either of the short-wavelength-sensitive opsins (SWS1, SWS2) with MSP. Relative expression of *SWS1* was the lowest of the four visual opsins, suggesting that if SWS1 photoreceptors exist in leopard frogs, they may be rare and/or small and thus difficult to detect and measure with MSP. In both *X. laevis* and *L. catesbeianus*, SWS1 opsin was localized to a subset of single cone photoreceptors using immunohistochemistry, while SWS2 was exclusive to SWS2 “green” rods [57, 58, 94, 95]. This is in contrast to salamanders, which can have both SWS1 and SWS2 cones in addition to SWS2 rods [96, 97]. Using MSP, *L. catesbeianus* was found to have blue-sensitive cones with the same λ_max_ (433 nm) as the SWS2 rods [62], which when combined with the immunohistochemistry data [57, 94] suggests that the SWS1 and SWS2 visual pigments may have converged on the same absorbance spectrum in this species [56]. Blue-sensitive cones have not been found with MSP in *X. laevis* [98], but the absorbance spectra of the SWS1 and SWS2 visual pigments were measured in vitro (with the A_1_ chromophore) and found to differ by almost 10 nm (425 and 434 nm, respectively [58, 95]). It is also possible that frogs co-express SWS1 with other visual pigments, as occurs in salamanders and several other vertebrate groups [97, 99]. This could further explain the difficulty in detecting SWS1 in southern leopard frogs with MSP. Additional studies are clearly needed to further resolve the photoreceptor complements of frogs and how they vary across taxa.

Differential expression of phototransduction genes can also be an important factor in adaptation to dim- and bright-light environments but has been less extensively studied [65]. The bright-light (cone) and dim-light (rod) photoreceptors express partially distinct subsets of phototransduction genes, and the differences in the function and abundance of the encoded proteins form an important part of the basis for the difference in physiology between them [100]. We found that 29 of the 34 phototransduction genes were significantly differentially expressed between tadpoles and juveniles, with cone genes upregulated in tadpoles and rod genes upregulated in juveniles. This finding is consistent with adaptation to the primarily diurnal habits of tadpoles and the primarily nocturnal habits of juveniles and adults [49, 51]. The higher relative expression levels of rod genes in juveniles suggests that the proportion of rods to cones is higher in juveniles, although the size of rods and/or the expression of rods genes within individual rods may also contribute to the increased expression levels we observed. This also agrees with the observation that in at least some frog species (*Rana temporaria*, *Xenopus laevis*), the cones develop earlier and faster than the rods, based on histological, behavioral, and electrophysiological evidence [101]. In *Lithobates pipiens*, however, rods and cones may begin development at the same time based on transmission electron micrographs from which rods and cones were distinguished based on minor ultrastructural differences in outer segment disc morphology [102]; the reliability of this particular feature for distinguishing among rods and cones is unclear. As far as we are aware, broader analysis of photoreceptor proportions has only been performed in adult leopard frogs where in *L. pipiens*, it was reported that 50% of cells were RH1 rods, 15% SWS2 rods, 35% cones [64], and in adult *X. laevis* in which 53% of cells were rods (only ~ 3% of which were green rods [59]). Future studies that analyze ontogenetic variation in photoreceptor abundances (and those of other retinal cell types), especially when combined with differential expression analyses, will be needed to further explore this issue.

### Photoreceptor spectral sensitivity variation in closely related leopard frog species

Our *Lithobates sphenocephalus* MSP results are fairly similar to spectral absorbances previously reported for the closely related northern leopard frog (*L. pipiens* [53, 54]) but with some notable differences. Estimates for RH1 rods across species only differed by 1 nm in tadpoles (λ_max_ = 526 vs 527 nm [both inferred to be primarily A_2_-based] for *L. sphenocephalus* and *L. pipiens*, respectively). The protein sequences for RH1 are nearly identical between the two species and so the resulting visual pigments are expected to have very similar absorbances. In adult *L. sphenocephalus*, we were able to distinguish two potentially distinct populations of RH1 rods: the more numerous class had a mean λ_max_ of 505 nm with an A_1_ best-fit, while the less numerous had a λ_max_ of 501 nm with an A_2_ best-fit. Interestingly, Liebman and Entine [53] also classified two RH1 rod subtypes in adult *L. pipiens*: the more numerous with λ_max_ at 502 nm and the rarer with λ_max_ as high as 507 nm, both thought to be A_1_ based. The higher λ_max_ of some rods could be due to a mixture of a small amount of A_2_-based pigment with the A_1_-based pigment in a subset of rod cells. Liebman and Entine [53] argued against this possibility because they found both rod types had the same A_1_ bleaching intermediates in *L. pipiens*, but it is unclear what other mechanism could account for this observation. Alternatively, the two RH1 rod populations could be due to the presence of two RH1 alleles that encode spectrally distinct RH1 opsin proteins, but we found no evidence to support additional RH1 (or other visual opsin) alleles based on our de novo assembled eye transcriptome data. Thus, we tentatively conclude the second group of RH1 rods in our MSP dataset is most likely due to variation in the proportion of A_2_-based pigment, which is further supported by the variable levels of *CYP27C1* expression we found for the juvenile individuals. The presence of two RH1 rod populations could also be related to spatial variation in the ratio of A_1_- to A_2_-based visual pigments across the retina, as found in adult bullfrogs (*L. catesbeianus* [37]). We cannot rule out that the differences are due to error associated with MSP measurements and curve-fitting, especially when both sets of rods have the same mean λ_max_ when fit with A_1_ templates (505 nm). Quantitative studies of chromophore content would allow more conclusive differentiation among these potential explanations.

Changes in spectral sensitivity of SWS2 rods showed opposite trends across life stages in southern and northern leopard frogs [53]. In tadpoles, *L. sphenocephalus* had the lower λ_max_ (433 nm compared to 438 nm in *L. pipiens*), whereas in adults, *L. sphenocephalus* had the higher λ_max_ (437 nm compared to 432 nm in *L. pipiens*). Unfortunately, our SWS2 rod estimate from the *L. sphenocephalus* tadpole was based on only a single noisy scan, so our estimates of λ_max_ and chromophore type may be inaccurate. However, our results do support a small (~ 5 nm) red shift for the adult SWS2 rods in *L. sphenocephalus* relative to *L. pipiens*. For the tadpole LWS cones, we found a similar red shift between species (λ_max_ = 626 nm in *L. sphenocephalus* vs 620 nm [both inferred to be primarily A_2_-based] in *L. pipiens*). In adults, the 579 nm cone we identified in *L. sphenocephalus* is similar to the A_1_-based 575 nm LWS cones reported for *L. pipiens* [53], but again slightly red-shifted. This implies that the second type of LWS cone we found (λ_max_ = 603 nm) may be the result of a small amount of A_2_ pigment mixed with A_1_-based pigment, also resulting in a red shift. Unfortunately, protein sequences for *L. pipiens* SWS2 and LWS are not available for comparison to determine if changes to the protein sequence are likely to contribute to the observed differences in λ_max_. Alternatively, these differences may be the result of *L. sphenocephalus* maintaining higher A_2_ chromophore levels in adults than *L. pipiens*, specifically in the LWS cones and SWS2 rods. Differences in chromophore proportions among photoreceptor types have been found previously in sticklebacks [103], but it is unclear whether this occurs in other vertebrates. Overall, these results suggest there may be a large amount of unappreciated variation in frog photoreceptor complements and spectral sensitivities that requires further investigation.

### Lens crystallin expression and estimated refractive index of the lens shifts across ontogeny

Previous studies of frog lens crystallin proteins found predominantly γ-crystallins (CRYG) in tadpoles and α- and β-crystallins in adults (CRYA and CRYB) [28, 47, 48], which is partly consistent with our observations of crystallin gene expression. Although the combined level of α- and β-crystallin gene expression increased relative to γ-crystallins in juveniles, the expression of β-crystallin genes actually decreased slightly. This is contrary to expectations based on protein studies in *L. catesbeianus* [47, 104]. However, it is possible that with further growth a similar shift would be observed in β-crystallin gene expression in *L. sphenocephalus* considering that changes in crystallin composition have been linked to increases in lens diameter rather than with metamorphosis [48]. Beyond the overall changes in α-, β-, and γ-crystallin expression, our findings suggest a more complicated scenario of crystallin usage at the gene level. Different β- and γ-crystallin genes were upregulated in tadpoles versus juveniles indicating turnover in both of these types of crystallins.

One of the primary roles of crystallins is to provide a high refractive index to the lens [105]. Experimental and computational studies have estimated refractive index increments for multiple crystallins, and α-crystallins were found to have the lowest refractive indices, followed by β-, and then γ-crystallins, which have exceptionally high values compared to other proteins [105, 106]. Our estimates of refractive increments for the leopard frog ubiquitous crystallins agreed with this general trend. Consequently, we predict that the increased expression of *CRYAB* (the encoded crystallin of which had the lowest estimated refractive index), and decreased expression of γ-crystallin genes in juveniles would reduce the overall refractive index of the juvenile lens relative to the tadpole lens. This change in refractive index across ontogeny likely reflects the need to avoid overfocusing (myopia/nearsightedness) when juveniles transition to vision in air, where the cornea provides substantial refractive power. This explanation is further supported by Mahendiran et al. [72] who found aquatic vertebrates (*X. laevis* and zebrafish) generally had crystallins with higher refractive increments than terrestrial mammals.

We found that the two β-crystallins encoded by genes upregulated in tadpoles (CRYBA1, CRYBB1) had higher refractive increments than the β-crystallin encoded by a gene upregulated in juveniles (CRYBB2), suggesting that a similar shift in the refractive increments of crystallins could contribute to a change in the refractive index of the lens across ontogeny in frogs with aquatic larval and terrestrial adult life stages. However, it should be noted that the pattern within γ-crystallins was not as clear and that when accounting for relative expression levels (as a rough approximation for protein abundances), we found that the mean refractive increment of the crystallin composition in juveniles was only slightly less than that in tadpoles. It has been proposed that the shift in crystallin composition from primarily γ-crystallin to α- and β-crystallins serves to maintain the refractive index of the frog lens during growth, while an increased hydration of the lens, along with the change in lens shape, may be responsible for the decreased power of the lens in the transition to vision in air [46]. The difference in γ-crystallin usage could also be related to a change in lens hydration. Zhao, et al. [107] found that zebrafish γ-crystallins exhibited extremely low degrees of hydration consistent with their role in high refractive index aquatic lenses. This was contrasted with average and low degrees of hydration of different mouse and human γ-crystallins. A shift in γ-crystallin usage in frogs that transition from aquatic to terrestrial habitats could facilitate a change in lens hydration, but this remains to be tested. However, these results highlight that additional studies are needed both to examine frog lens crystallins more directly, and to examine how they turnover across the full range of ontogeny, to better understand how the lens has adapted in response to different light environments.

Two taxon-specific crystallins have been identified in frog lenses, but the specific roles they play in the function of the lens have not been studied. ρ-crystallin (originally referred to as ε-crystallin) was first found in *Rana temporaria* and later in *L. catesbeianus* [67, 108], while ζ-crystallin was identified in the lenses of the hylid frogs *Hyla japonica*, *Litoria infrafrenata*, and *Phyllomedusa sauvagei* [108, 109]. We found significant differential expression of both ρ- and ζ-crystallin genes in leopard frog eyes; however, the very low relative expression of ζ-crystallin suggests that it does not function as a lens crystallin in this species. A third taxon-specific crystallin, α-enolase (ENO1, τ-crystallin) has mixed reports regarding its presence in the lenses of *Bufo* toads [70, 71]. We found that *ENO1* was differentially expressed across life stages and expressed at moderate levels, but like most other taxon-specific crystallins, ENO1 has varied enzymatic functions and broad tissue expression [110]; thus, its role as a lens crystallin in leopard frogs requires further investigation.

## Conclusions

We found high levels of decoupling of gene expression between aquatic tadpole and terrestrial juvenile southern leopard frogs consistent with the adaptive decoupling hypothesis. The degree of decoupling was even greater among visual genes, suggesting that adaptive decoupling may have played an important role in the evolution and adaptation of anuran visual systems. Specifically, our results highlight expression differences in a range of visual genes, including chromophore usage, visual opsin, and lens crystallin genes that likely underlie observed morphological and physiological changes through metamorphosis and corresponding adaptive shifts to improve visual ability in aquatic versus terrestrial light environments. We also found evidence that light/dark exposure has a significant effect on the expression of a much smaller, but similar, set of visual genes. These results set the stage for investigating adaptive decoupling and differential expression of visual genes across a broader ecological sampling of larval and metamorphosed amphibians and further investigating the plasticity of visual gene expression in vertebrates.

## Methods

### Study animals

Twelve southern leopard frogs (*Lithobates sphenocephalus* [=*Rana* (*Pantherana*) *sphenocephala* [111]]; six tadpoles and six juveniles) were obtained from a single wild population in Arlington, TX in May and June 2018 (*-*97.101168, 32.792202; Texas Parks and Wildlife Scientific Research Permit No. SPR-0814-159; UTA IACUC Protocol A17.005) (Additional file 1: Table S1). Animals were captured within a few days of one another and held for a few hours in the laboratory prior to the experimental treatment. The tadpoles were Gosner stages 25–38 and juvenile snout-vent lengths (SVL) were 24.77 mm to 34.59 mm (less than 51 mm, which is the minimum SVL leopard frogs are reported to reach sexual maturity at ([112]; Additional file 1: Table S1). Three tadpole and three juvenile specimens were exposed to ambient light in the laboratory (~ 3 h) or complete darkness (i.e., dark adapted) for 12 h prior to euthanasia (via immersion in a solution of MS222). The two light treatments each included one early stage (25–27) and two later stage (30–34 in the light treatment and 35–38 in the dark treatment) tadpoles (Additional file 1: Table S1). One whole eye from each specimen was extracted and placed in RNAlater (Ambion) for at least 24 h at 4 °C to allow the RNALater to saturate the cells, prior to freezing and storage at − 80 °C until use. For the dark-adapted specimens, eyes were dissected in the dark, placed in RNAlater, and then wrapped in foil to keep them in the dark during the entire process. An additional tadpole (not staged) and an adult (SVL 66.6 mm) were collected in April and June 2019 from the same location for microspectrophotometry (MSP). Due to sample availability at the time the MSP samples were collected, we were unable to obtain a juvenile individual. However, our goal was to compare the spectral sensitivity of aquatic tadpoles to terrestrial post-metamorphic frogs, and thus, the use of a juvenile or adult is not expected to affect the interpretation of the results because changes in spectral sensitivity occur during metamorphosis (for a review, see [34]), and both are post-metamorphic and terrestrial. Further, a previous study that compared spectral sensitivity between juvenile and adult frogs found no difference in spectral sensitivity [82]. Voucher specimens are accessioned at the Amphibian and Reptile Diversity Research Center at UT Arlington and Smithsonian Institution’s National Museum of Natural History (Additional file 1: Table S1).

### Transcriptome sequencing and assembly

Total RNA was extracted from whole eyes using the Promega Total SV RNA Extraction kit (Promega). Tissue was homogenized in the prepared lysis buffer using the Qiagen Tissuelyzer (10 min at 20 Hz). Messenger RNA library construction was performed using the Kapa HyperPrep mRNA Stranded with Riboerase kit (Roche). Each indexed sample was pooled in equimolar amounts and sequenced on one lane of a HiSeq4000 with paired end 150 bp reads by Novogene.

Several quality control steps were employed to improve the quality and efficiency of the transcriptome assembly*.* Erroneous k-mers were removed from raw reads with rCorrector [113] and a custom script [114]. Adapters and low-quality bases (*q* < 5) were removed with TrimGalore! v0.6.5 [115], which implements Cutadapt [116]. Read pairs shorter than 36 bp after trimming were discarded, as were unpaired reads. Ribosomal RNA reads were removed by mapping reads with Bowtie2 [117] against the SILVA database [118]. Quality of processed reads was assessed with FastQC (http://www.bioinformatics.babraham.ac.uk/projects/fastqc/). A de novo reference transcriptome was assembled using Trinity v2.8.5 [119] incorporating all paired reads from each of the 12 samples following the standard protocol. This assembly was subsequently redone removing one of the samples that was found to be an outlier in a principal component analysis of differential expression (see below). Alignment summary metrics were calculated using Trinity [119]. Read support for the assembly was determined by mapping the reads back to the assembly using Bowtie2 and completeness was assessed using BUSCO (Benchmarking Universal Single-Copy Orthologs) v3.0.2 [120] with the Tetrapoda dataset. Because de novo assemblies, especially with a large number of samples, are expected to produce many transcripts that are spurious, do not represent functional, protein-coding transcripts, or have very low read support, we reduced the initial set of Trinity transcripts to a “best” set of transcripts using a modified version of the “Trinity best transcript set” pipeline (https://github.com/trinityrnaseq/trinity_community_codebase/wiki/Trinity-best-transcript-set) available at (https://github.com/ryankschott/Best_Transcript_Set_Updated). This pipeline uses TransDecoder (https://github.com/TransDecoder), InterProscan [121], and cd-hit [122] to identify protein-coding transcripts and reduce the initial transcripts to a “best” set containing those transcripts.

### Differential expression and gene ontology enrichment analyses

Abundances of the reduced transcript set were quantified using Salmon [123] and scripts included with Trinity. Differential expression was estimated with DESeq2 [124] using a generalized linear model with a negative binomial distribution and a multifactor design accounting for both life stages (tadpole vs juvenile) and treatment (light vs dark exposure)*.* An adjusted *P*-value (padj) of < 0.05 was used as the significance cut-off for differential expression. For data visualization and clustering, raw counts were transformed using the regularized logarithm (rlog), and log_2_ fold changes (LFC) were shrunk using the apeglm method [125]. A principal component analysis (PCA) of rlog transformed counts was used to evaluate variation among the samples and to identify potential outliers. One of the juvenile, dark-exposed samples was identified as an outlier and removed from further analyses (Additional file 1: Fig. S1). Transcripts were annotated using BLASTn against the NCBI nucleotide (nt) database. To assess changes within the eye transcriptome that are specific to the visual system, we generated a second dataset for differential expression analyses using a curated subset of 170 visual gene coding sequences based on sets of visual genes curated in amniotes [76, 126], which we adapted for use in anurans using NCBI GenBank and BLAST searches to identify and annotate the coding sequences in *Xenopus*, *Nanorana parkeri*, and the *L. sphenocephalus* eye transcriptome. The genes included in the list are focused on those with exclusive, or at least primary functions, in the initial stages of vision in the eye, and especially within the photoreceptors, including opsins, phototransduction, visual cycle, lens, and eye, retina, and photoreceptor development. For a full list of genes, their general functions, and source links, see Additional file 5. Reads were quantified against this reference using Salmon, and differential expression was estimated following the same approach outlined above. The same juvenile, dark-exposed sample was also identified as an outlier in the visual gene dataset and excluded from subsequent analyses (Additional file 1: Fig. S3). To compare relative expression among groups of genes, we used the Trinity pipeline to calculate cross-sample normalized (TMM) expression values [127]. To compare the proportion of cone and rod phototransduction genes upregulated and downregulated in tadpoles and juveniles, we used a Z-pooled exact test implemented in the R package “Exact” [128] with the binomial model, fixed row margins, and the null hypothesis that the difference in the proportion of upregulated rod and cone phototransduction genes was equal in tadpoles and juveniles.

We annotated the reduced set of transcripts using DIAMOND [129] blastx search with the “sensitive” option against the *Xenopus tropicalis* (v9.1) ENSEMBL database. We obtained GO terms for the best hit ensemble protein ID that each transcript was annotated with using the Uniprot Retrieve/ID mapping tool [130]. GO enrichment analyses were performed using TopGO for the biological process gene ontology with the combined gene elimination and weighting algorithm (weight01) and Kolmogorov-Smirnov testing (KS) [131]*.*

### Microspectrophotometry

Microspectrophotometry was conducted on eyes from one tadpole and one adult (see above) following protocols described in previous studies [132, 133]. After at least 2 h of dark adaptation, animals were euthanized with MS-222 and the eyes enucleated under dim red light. Eyes were hemisected, the cornea and lens isolated, and the retinas carefully removed from the pigment epithelium under hypertonic buffer (pH 7.2 supplemented with 6% sucrose). Pieces of retina were macerated, sandwiched between two coverslips edged with silicone grease, and placed on the stage of a computer-controlled single-beam MSP [132]. Our focus was on obtaining measurements from the RH1 “red” rods, but absorbance spectra were obtained for clearly identified outer segments of multiple photoreceptor cell types from 750 to 350 nm, and back again from 350 to 750 nm, with a wavelength accuracy of ~ 1 nm [132]. Visual pigment λ_max_ was determined by template fitting using previously described methods [132]. Briefly, a Gaussian function was fit to the top 40 data points at 1 nm intervals and differentiated to establish the peak wavelength. The spectrum was normalized to this absorbance value and template fit to either A_1_ or A_2_ standard data [55] using the method of MacNichol [134]. Spectra were fit to pure A_1_ and A_2_ templates as a methodological decision for repeatability in template fitting and not an assumption that the pigments were pure A_1_ or A_2_. Template fitting alone is not the best determinant of A_1_ or A_2_ status for noisy data such as those from the very small outer segments of amphibian tadpoles and adult cones. However, if the calculated λ_max_ was greater than 580 nm, it was assumed that A_2_ must be present [63]. Calculated λ_max_ values are accurate to ±1.0 nm and are reported here to the nearest whole integer.

### Protein refractive index increment estimation

Protein refractive index increments (*dn*/*dc*) were estimated for the predicted leopard frog lens crystallin proteins using the method of Zhao, Brown and Schuck [135] as implemented in the SEDFIT v16.1c software [136]. The protein refractive index increment defines how much a given concentration of a protein contributes to the overall refractive index of the solution, which in the case of lens crystallin proteins equates to how much they will contribute to the refractive index of the lens. The method of Zhao and colleagues [135] uses a biophysical computational model to estimate refractive index increments based on the amino acid composition of the protein and the refractivities of those amino acids as calculated by McMeekin et al. [137]. Estimates were made assuming 589 nm light at 25 °C and a solvent refractive index of 1.3340 following the protocol established by Zhao, Brown, and Schuck [135].

## Supplementary Information

**Additional file 1: Figure S1.** Principal components analysis (PCA) plot of rlog transformed counts from the A) transcriptome-wide and B) vision gene coding sequence analyses illustrating the outlier juvenile, dark exposed sample. **Figure S2.** Enrichment of Gene ontology (GO) terms. **Figure S3.** Principal components analysis plot of rlog transformed counts of visual gene coding sequences. **Figure S4.** Spectral sensitivity of photoreceptors detected through microspectrophotometry in (A) one tadpole and (B) one adult *Lithobates sphenocephalus*. **Figure S5.** Examples of raw microspectrophotometry data and raw Gaussian fits (coloured and transparent) and Govardovskii et al. [55] template fits (black and non-transparent) for each cell and pigment type found in the tadpole (**A**–**C**) and adult (**D**–**G**) *Lithobates sphenocephalus*. **Figure S6.** Examples of raw microspectrophotometry data and raw Gaussian fits (cyan) and Govardovskii et al. [55] template fits (black) for RH1 ‘red’ rods in *Lithobates sphenocephalus* adults where the A_2_ template (solid) was a better fit than the A_1_ template (dashed). **Figure S7.** Expression profiles of significantly differentially expressed cone and rod phototransduction genes between tadpoles and juveniles. **Figure S8.** Comparison of averaged cross-normalized expression levels (TMM) for each major crystallin type. **Figure S9.** Expression profiles of taxon-specific lens crystallin genes that have not specifically been identified in frogs and that differ substantially between tadpole and adults (adjusted *P*-value < 0.05; Additional file 6). **Figure S10.** Comparison of averaged refractive increment index (*dn*/*dc*) for each major crystallin type. **Table S1.** Sample information and read counts for the frogs used in the RNA-seq analyses. **Table S2.** Summary data from fits of individual photoreceptor scans from one *Lithobates sphenocephalus* tadpole (*n* = 41) and one adult (*n* = 33) to visual pigment templates from Govardovskii et al. [55]. **Table S3.** Cross-normalized expression values (TMM) and computationally estimated refractive index increment (*dn*/*dc*) values for the ubiquitous and known frog taxon-specific specific crystallins.

**Additional file 2. **Results of BUSCO analyses on each of the three *de novo* transcriptome assemblies.

**Additional file 3.** Results of DESeq2 analyses on the reduced transcriptome.

**Additional file 4.** Results of GO enrichment analysis with TopGO.

**Additional file 5.** Description of genes included in the visual gene dataset.

**Additional file 6.** Results of DESeq2 Analyses on the visual gene dataset.

**Additional file 7.** Results MSP measurements and curve-fitting on each individual photoreceptor cell.

**Additional file 8. **Cross-normalized expression values for the visual gene dataset and *dn/dc* calculations for the lens crystallins.

## Data Availability

All data generated or analyzed during this study are included in this published article, its supplementary information files and publicly available repositories. Raw sequence data were deposited in the NCBI under BioProject PRJNA839794 (see Additional file 1, Table S1 for individual accession numbers). Processed sequence data and the transcriptome assemblies are available in the Zenodo dataset [52]. Differential expression and MSP data are available in the supplementary information files and the Zenodo dataset [52]. Analysis scripts are available on Github [138].

## Abbreviations

BUSCO: Benchmarking Universal Single-Copy Orthologs
A_1_: Retinal
A_2_: 3,4-Didehydroretinal
*dn*/*dc*: Protein refractive index increments
GO: Gene ontology
LFC: Log_2_ fold change
MSP: Microspectrophotometry
PC: Principal component
PCA: Principal components analysis
rlog: Regularized logarithm
SVL: Snout-vent length
TMM: Cross-normalized expression values (trimmed mean of *M*-values)
