# Supplementary material for: Transcriptomic evidence for visual adaptation during the aquatic to terrestrial metamorphosis in leopard frogs
Source: BMC Biol. 2022 Jun 28;20:138. doi: 10.1186/s12915-022-01341-z (PMC9238225; doi:10.1186/s12915-022-01341-z)
Supplement: Supplementary file 1 — Additional file 1: Figure S1. Principal components analysis (PCA) plot of rlog transformed counts from the A) transcriptome-wide and B) vision gene coding sequence analyses illustrating the outlier juvenile, dark exposed sample. Figure S2. Enrichment of Gene ontology (GO) terms. Figure S3. Principal components analysis plot of rlog transformed counts of visual gene coding sequences. Figure S4. Spectral sensitivity of photoreceptors detected through microspectrophotometry in (A) one tadpole and (B) one adult Lithobates sphenocephalus. Figure S5. Examples of raw microspectrophotometry data and raw Gaussian fits (coloured and transparent) and Govardovskii et al. [55] template fits (black and non-transparent) for each cell and pigment type found in the tadpole (A–C) and adult (D–G) Lithobates sphenocephalus. Figure S6. Examples of raw microspectrophotometry data and raw Gaussian fits (cyan) and Govardovskii et al. [55] template fits (black) for RH1 ‘red’ rods in Lithobates sphenocephalus adults where the A2 template (solid) was a better fit than the A1 template (dashed). Figure S7. Expression profiles of significantly differentially expressed cone and rod phototransduction genes between tadpoles and juveniles. Figure S8. Comparison of averaged cross-normalized expression levels (TMM) for each major crystallin type. Figure S9. Expression profiles of taxon-specific lens crystallin genes that have not specifically been identified in frogs and that differ substantially between tadpole and adults (adjusted P-value < 0.05; Additional file 6). Figure S10. Comparison of averaged refractive increment index (dn/dc) for each major crystallin type. Table S1. Sample information and read counts for the frogs used in the RNA-seq analyses. Table S2. Summary data from fits of individual photoreceptor scans from one Lithobates sphenocephalus tadpole (n = 41) and one adult (n = 33) to visual pigment templates from Govardovskii et al. [55]. Table S3. Cross-normalized expression values (TMM [file 12915_2022_1341_MOESM1_ESM.pdf]

Additional File 1

Supplementary Figures and Tables for:

Transcriptomic evidence for visual adaptation during the aquatic to terrestrial metamorphosis in leopard frogs

Ryan K Schott[1,2*], Rayna C Bell[1,3], Ellis R Loew[4], Kate N Thomas[5], David J Gower[5], Jeffrey W Streicher[5], Matthew K Fujita[6]

[1]Department of Vertebrate Zoology, National Museum of Natural History, Smithsonian Institution, Washington DC, USA

[2]Department of Biology, York University, Toronto, Ontario, CAN

[3]Department of Herpetology, California Academy of Sciences, San Francisco, California, USA

[4]Department of Biomedical Sciences, Cornell University College of Veterinary Medicine, Ithaca, New York, USA

[5]Department of Life Sciences, The Natural History Museum, London, UK

[6]Department of Biology, Amphibian and Reptile Diversity Research Center, University of Texas at Arlington, Arlington, Texas, USA

*author for correspondence
Email: schott@yorku.ca

Contents:

Figures S1–S10

Tables S1–S3

Supplementary Figures

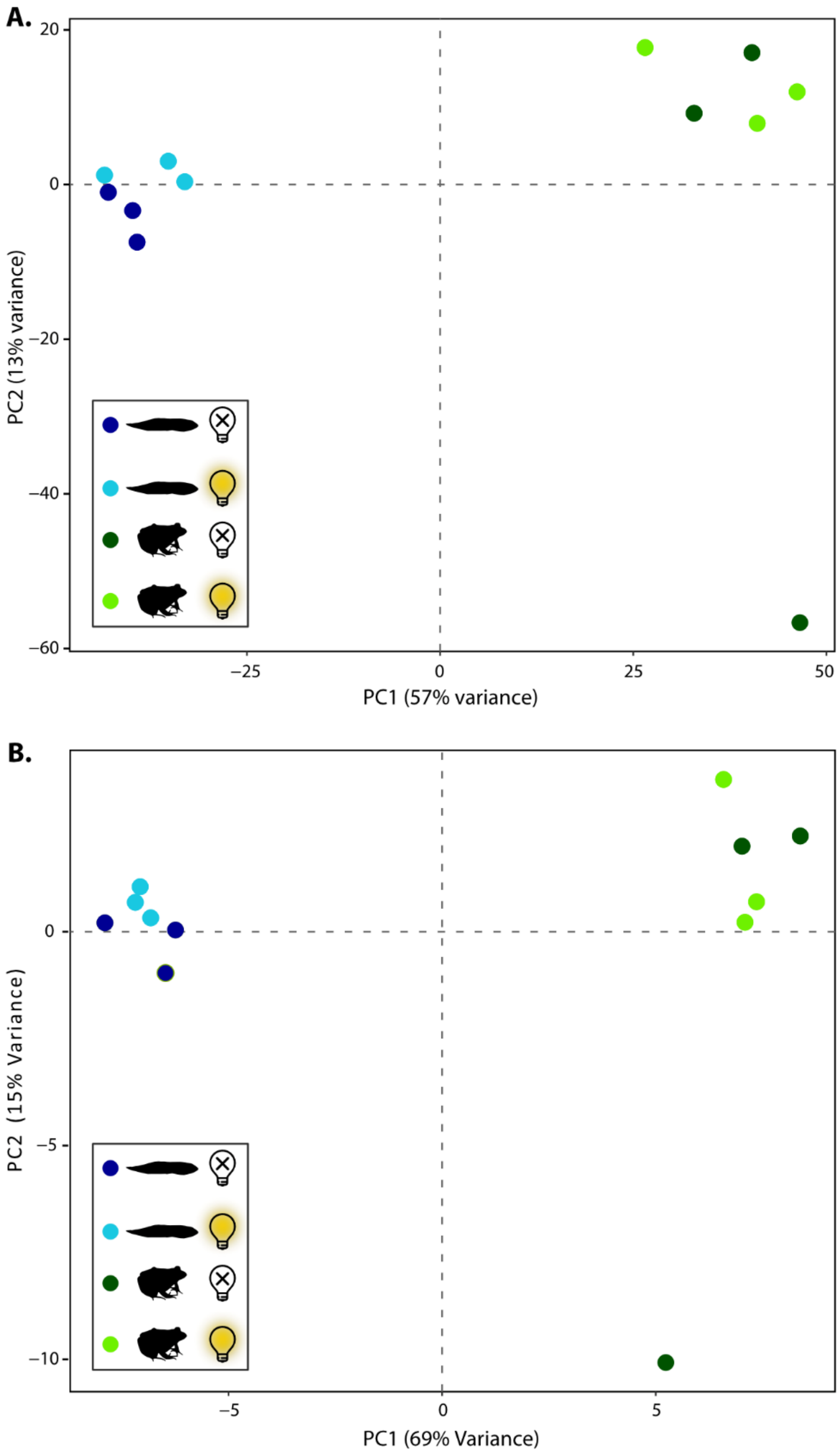

Figure S1. Principal components analysis (PCA) plot of rlog transformed counts from the **A**) transcriptome-wide and **B**) vision gene coding sequence analyses illustrating the outlier juvenile, dark

exposed sample. This sample was removed from subsequent analyses. Raw data are available in the Zenodo dataset.

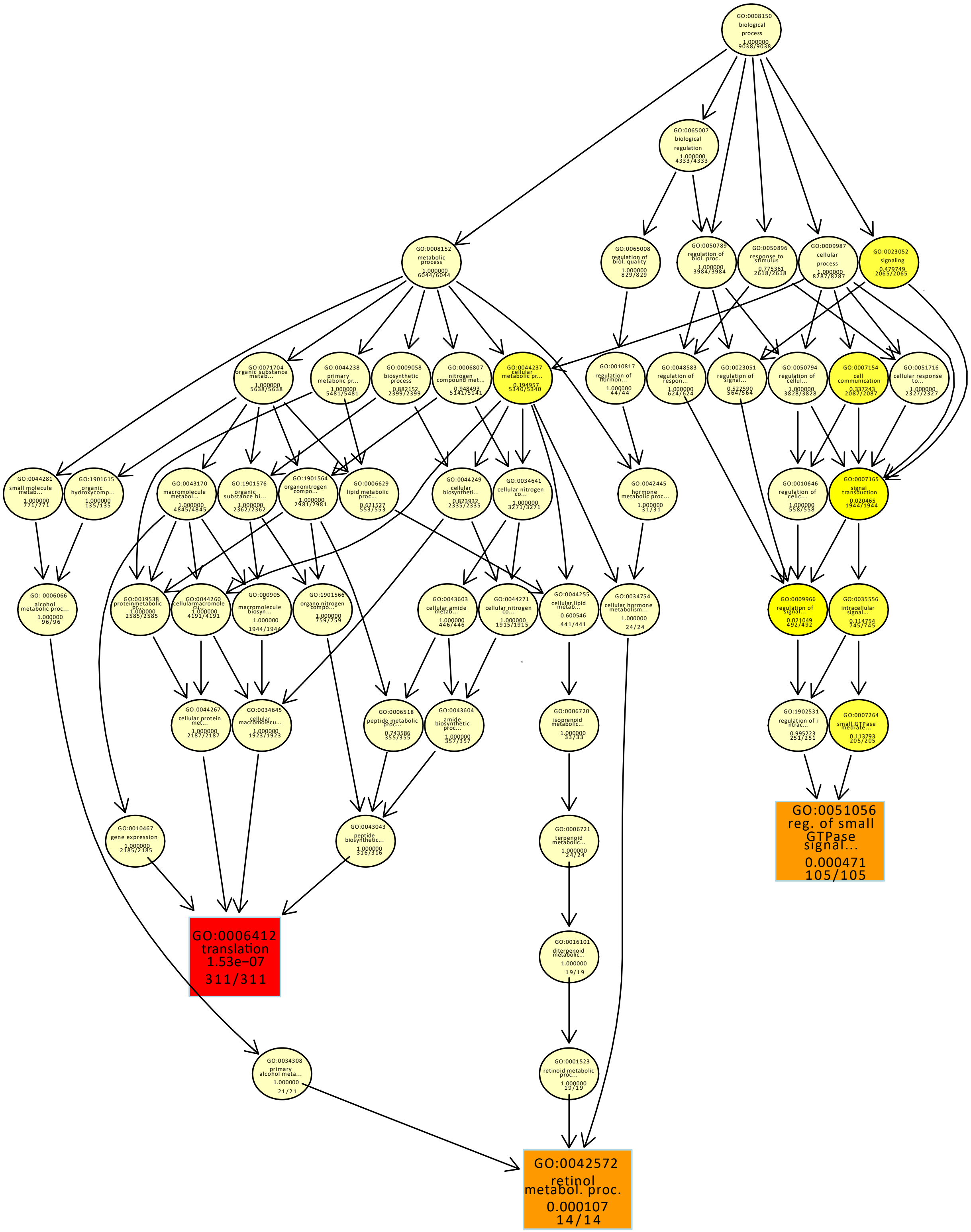

Figure S2. Enrichment of Gene ontology (GO) terms. GO terms, were based on annotation to the reduced set of transcripts using DIAMOND against the *Xenopus tropicalis* ENSEMBL database. GO enrichment analyses were performed using TopGO for the biological process gene ontology with the combined gene elimination and weighting algorithm (weight01) and Kolmogorov-Smirnov testing (KS).

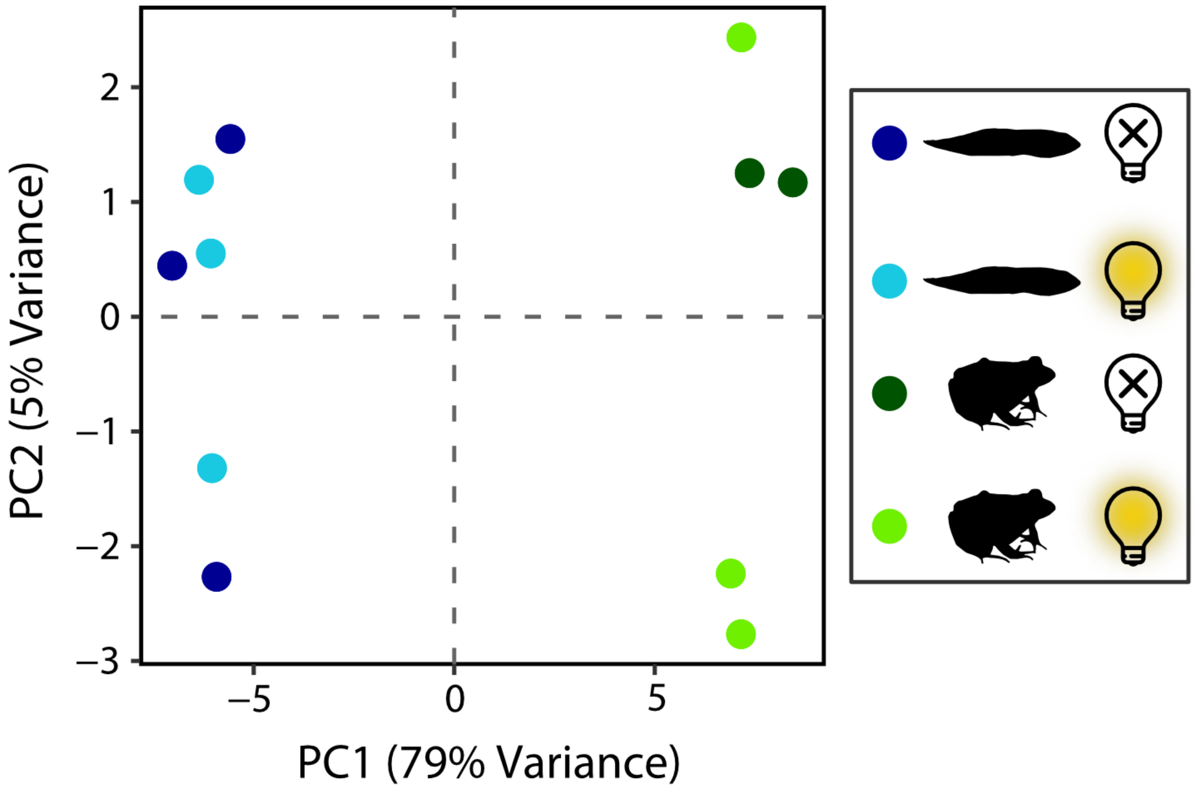

Figure S3. Principal components analysis plot of rlog transformed counts of visual gene coding sequences. The first principal component (PC1) accounting for 79% of the variance clearly separates juveniles and tadpoles. Light and dark exposure are not clearly separated by PC2, which accounts for only 5% of the variance. The PC values are available in Additional file 6.

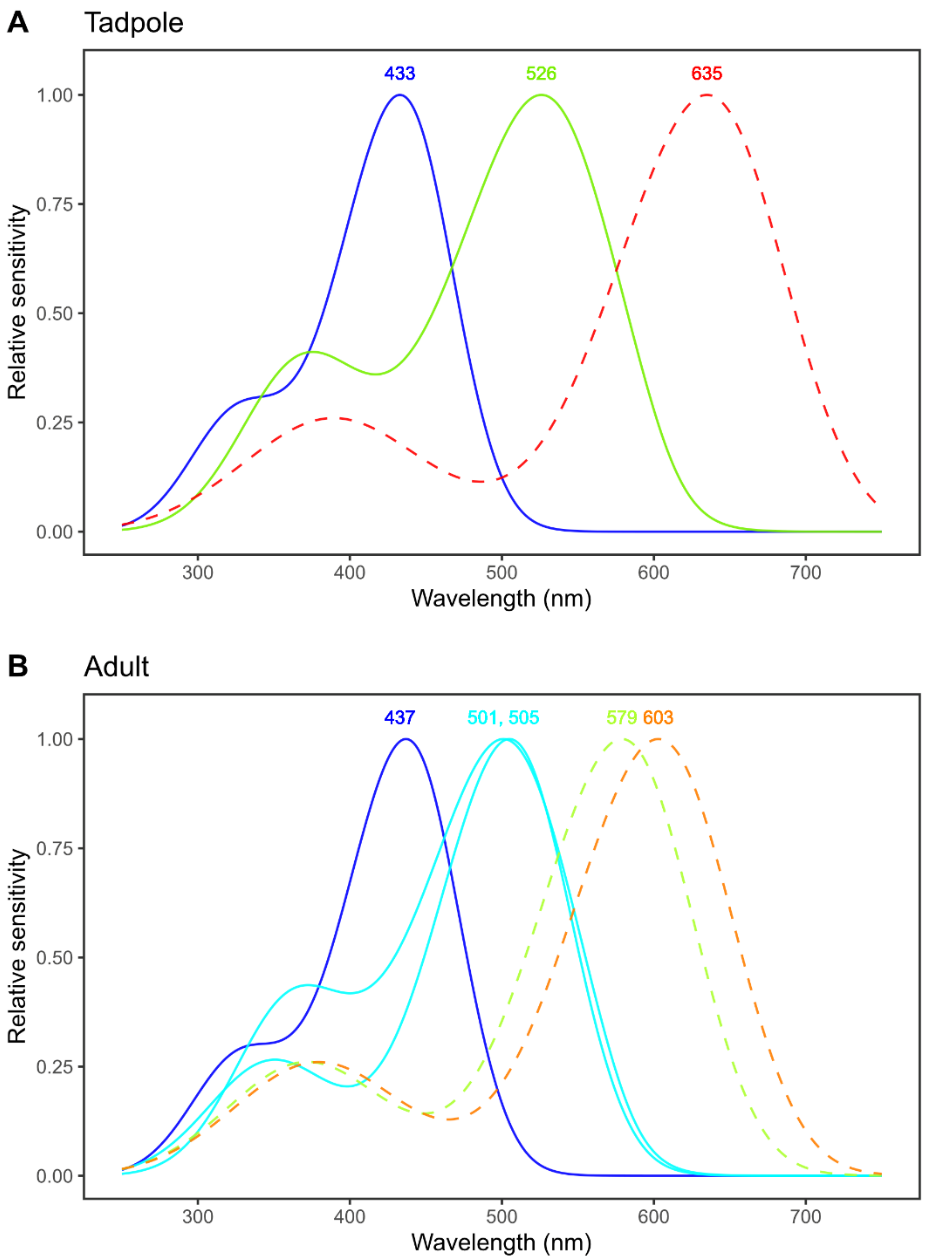

Figure S4. Spectral sensitivity of photoreceptors detected through microspectrophotometry in (**A**) one tadpole and (**B**) one adult *Lithobates sphenocephalus*. Govardovskii et al. (2000) visual pigment templates for the mean λ_{max} and best-fit chromophore type found for each pigment are displayed. Data from rods are shown with solid lines, and cones with dashed. MSP are available in Additional file 7 and in the Zenodo dataset.

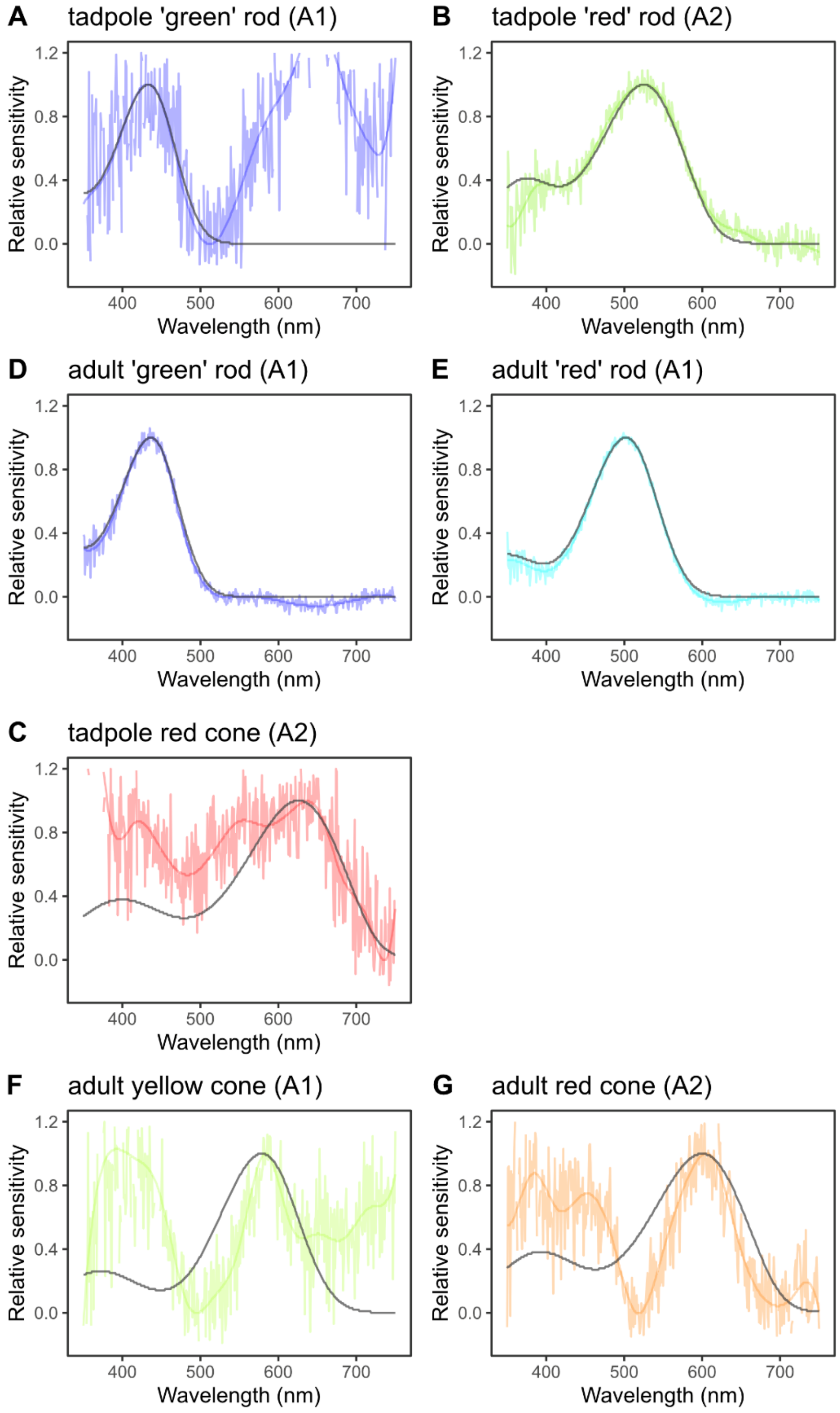

Figure S5. Examples of raw microspectrophotometry data and raw Gaussian fits (coloured and transparent) and Govardovskii et al. (2000) template fits (black and non-transparent) for each cell and pigment type found in the tadpole (**A–C**) and adult (**D–G**) *Lithobates sphenocephalus*. MSP are available in Additional file 7 and in the Zenodo dataset.

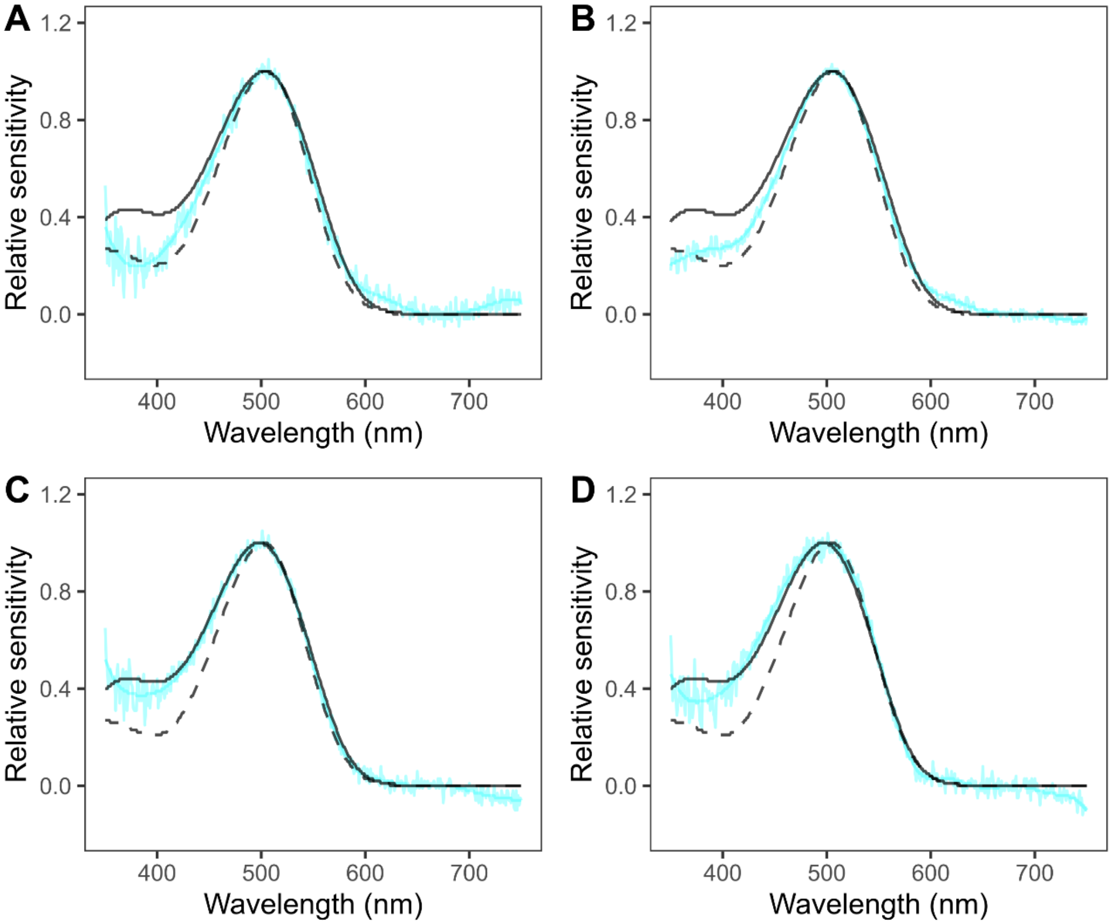

Figure S6. Examples of raw microspectrophotometry data and raw Gaussian fits (cyan) and Govardovskii et al. (2000) template fits (black) for RH1 'red' rods in *Lithobates sphenocephalus* adults where the A_2 template (solid) was a better fit than the A1 template (dashed). MSP are available in Additional file 7 and in the Zenodo dataset.

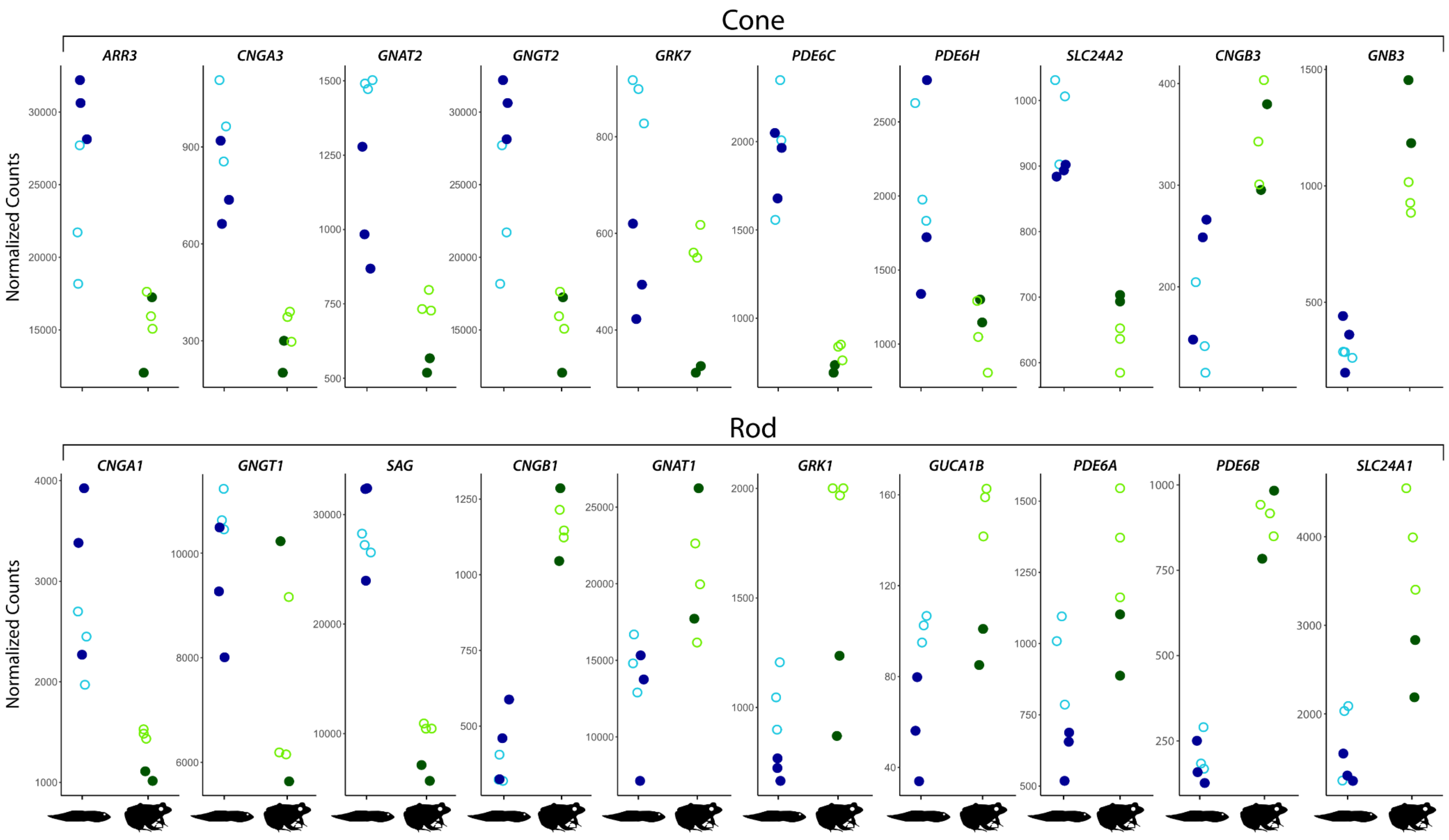

Figure S7. Expression profiles of significantly differentially expressed cone and rod phototransduction genes between tadpoles and juveniles. Cone genes (top row) tend to be upregulated in tadpoles, whereas rod genes (bottom row) tend to be upregulated in juveniles. Plots show normalized read counts (with an additional pseudocount of 0.5 to allow for log scale plotting) for each gene with light and dark exposed samples denoted by the open and closed circles, respectively. Normalized count data for each gene are found in Additional file 6.

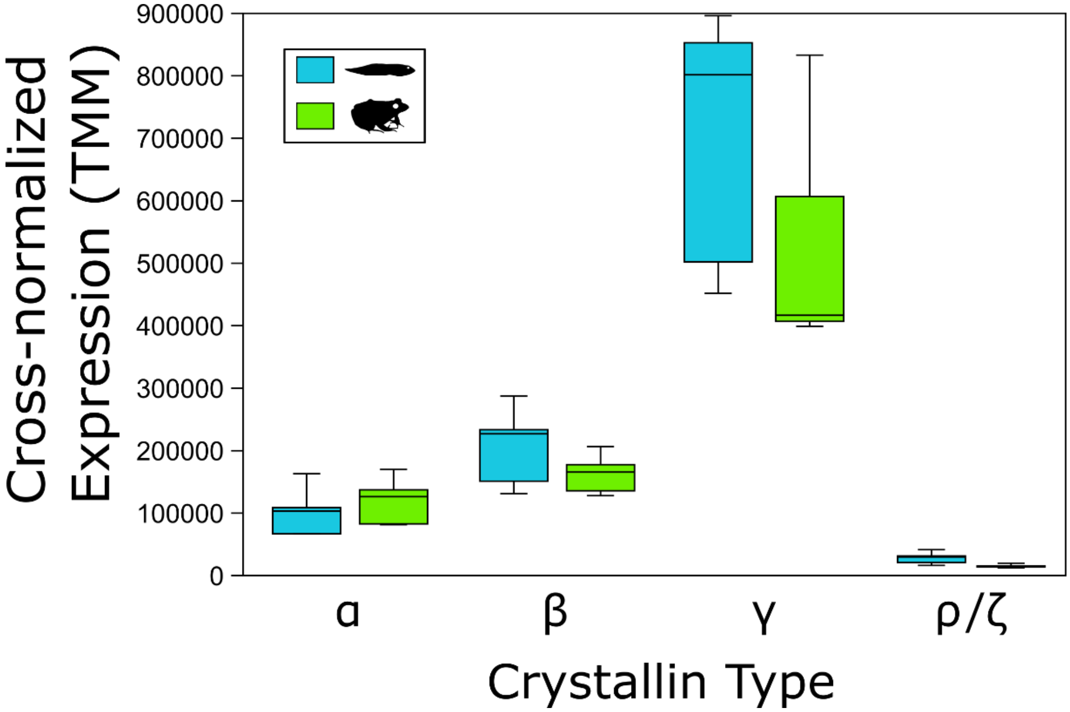

Figure S8. Comparison of averaged cross-normalized expression levels (TMM) for each major crystallin type. See also Table S3 and Additional file 8.

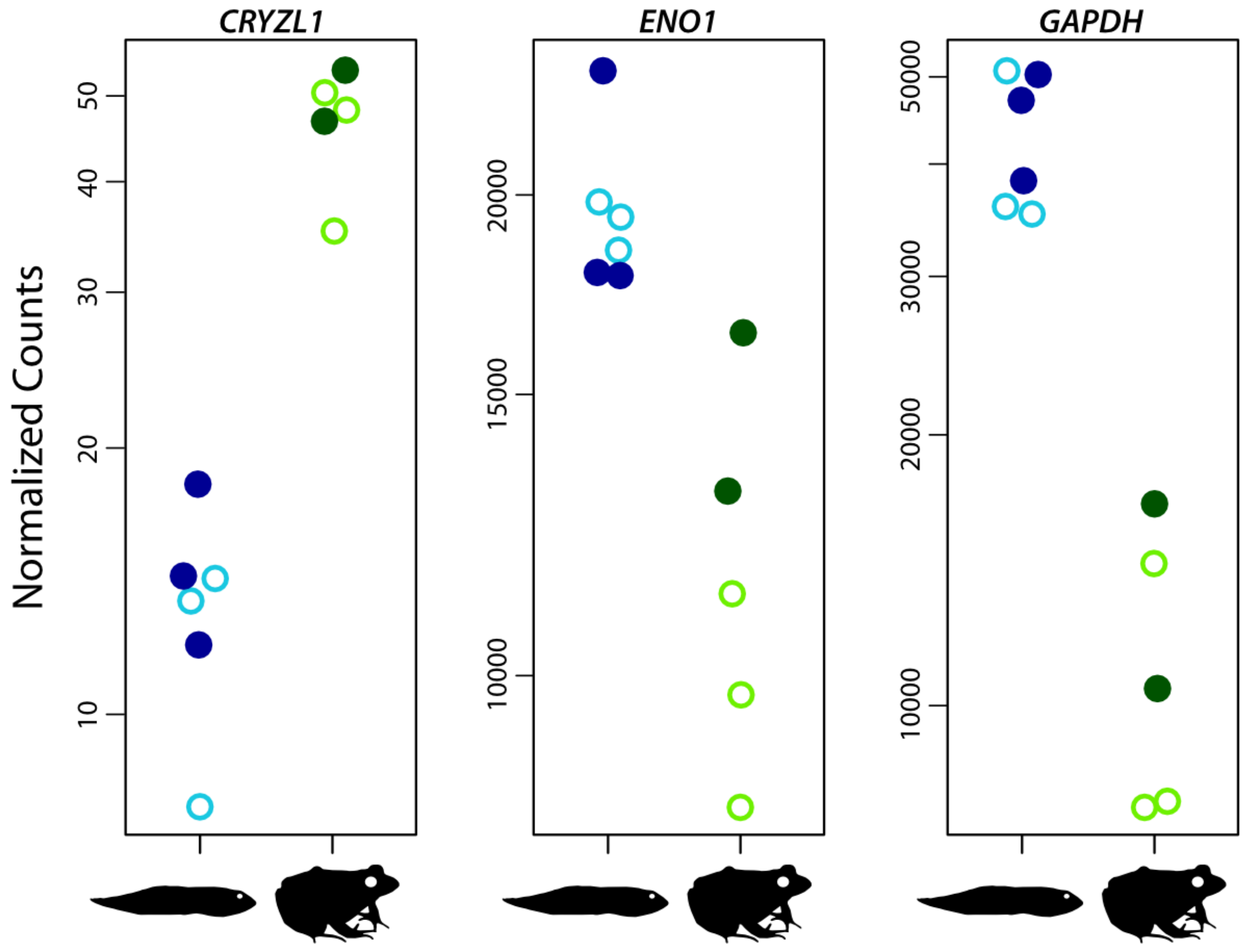

Figure S9. Expression profiles of taxon-specific lens crystallin genes that have not specifically been identified in frogs and that differ substantially between tadpole and adults (adjusted p-value < 0.05; Additional file 6). Plots show normalized read counts (with an additional pseudocount of 0.5 to allow for log scale plotting) for each gene with light and dark exposed samples denoted by the open and closed circles, respectively. Normalized count data for each gene are found in Additional file 6.

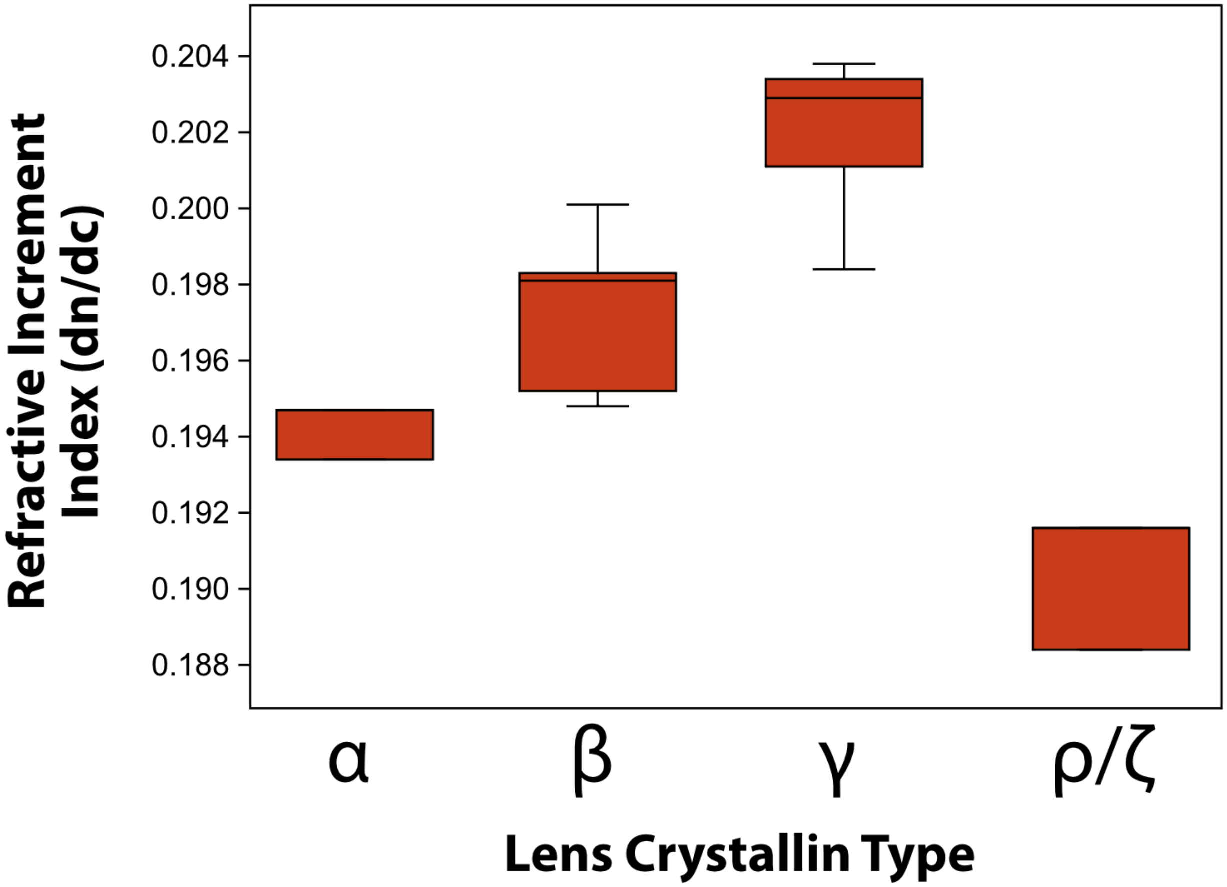

Figure S10. Comparison of averaged refractive increment index (*dn*/*dc*) for each major crystallin type. Values for *dn*/*dc* were computationally estimated using the SEDFIT program following Zhao et al. (2011). See also Table S3 and Additional file 8.

Table S1. Sample information and read counts for the frogs used in the RNA-seq analyses. Sample MKF956 was identified as an outlier and was dropped from the analyses.

Sample ID	BioSample Accession	Stage	Size (SVL) *or* Gosner Stage	Treatment	Raw Read Count	Cleaned Read Count
MKF939	SAMN28555757	Tadpole	25–26	Light	23477325	14685429
MKF940	SAMN28555758	Tadpole	30–34	Light	28579708	17590719
MKF941	SAMN28555759	Tadpole	30–32	Light	30730778	20181240
MKF943	SAMN28555760	Juvenile	34.59 mm	Dark	33986453	23719036
MKF944	SAMN28555761	Juvenile	25.64 mm	Light	30326831	20244107
MKF945	SAMN28555762	Juvenile	24.77 mm	Light	28315466	19763406
MKF946	SAMN28555763	Juvenile	26.30 mm	Light	31367780	22952832
MKF948	SAMN28555764	Tadpole	25–27	Dark	32122782	21163991
MKF949	SAMN28555765	Tadpole	35–38	Dark	40393269	25858753
MKF950	SAMN28555766	Tadpole	35–38	Dark	32209800	23040731
MKF955	SAMN28555767	Juvenile	29.70 mm	Dark	32457207	22374608
MKF956	SAMN28555768	Juvenile	32.74 mm	Dark	51226316	32448910

Abbreviations—**SVL**, snout vent length.

Table S2. Summary data from fits of individual photoreceptor scans from one *Lithobates sphenocephalus* tadpole (*n* = 41) and one adult (*n* = 33) to visual pigment templates from Govardovskii et al. (2000). Data were fit to templates based on 100% vitamin-A_1 and 100% vitamin-A2 chromophores, and the best fit is indicated, though λ_{max} and standard deviation of λ_{max} are reported for both template fits. Fits were averaged by spectral class (region of the visible spectrum where λ_{max} occurs) and cell type (rod or cone, identified by microscopy during MSP). The adult frog from which MSP data were collected is USNM 591950. Complete MSP results are in Additional file 7.

Age	Spectral Class	Cell Type	Visual Pigment	Best Fit	A1 λ_{max}	SD_{A1}	A2 λ_{max}	SD_{A2}	# Scans
**Tadpole**	blue	rod	SWS2	A1	433	–	431	–	1
	green	rod	RH1	A2	533	5.1	526	4.5	38
	red	cone	LWS	A1	635	3.5	626	0	2
**Adult**	blue	rod	SWS2	A1	437	1.5	442	2.6	8
	green	rod	RH1	A1	505	2.1	504	2.1	18
	green	rod	RH1	A2	505	2.1	501	3.9	4
	red	cone	LWS	A1	603	0	598	2.8	2
	yellow	cone	LWS	A1	579	–	579	–	1

Table S3. Cross normalized expression values (TMM) and computationally estimated refractive index increment (*dn*/*dc*) values for the ubiquitous and known frog taxon-specific specific crystallins. TMM values are averaged for the tadpole and juvenile replicates. A complete list of values can be found in Additional file 8.

Crystallin	TMM		*dn/dc*
	Tadpole	Juvenile	
**CRYAA**	94096.60	115676.48	0.1947
**CRYAB**	1943.32	17250.63	0.1934
**CRYBA1**	52622.86	33333.15	0.1983
**CRYBA2**	22825.81	16109.31	0.1981
**CRYBA4**	17840.00	14412.64	0.2001
**CRYBB1**	48380.53	27425.77	0.1952
**CRYBB2**	18514.59	44577.39	0.1948
**CRYBB3**	39951.36	45003.56	0.1956
**CRYG1**	36714.64	47688.54	0.2029
**CRYG2**	3552.10	562.33	0.2038
**CRYG3**	5027.47	25087.35	0.2031
**CRYG4**	73940.13	56876.37	0.2038
**CRYG5**	4935.20	3811.23	0.2022
**CRYG6**	308.35	1477.94	0.2023
**CRYG7**	98959.53	85580.08	0.2029
**CRYGB**	24381.80	60525.00	0.1994
**CRYGB2**	9397.70	8631.49	0.2011
**CRYGB3**	13.14	1333.23	0.1984
**CRYGD**	79878.25	72544.88	0.2037
**CRYGD2**	333928.82	214920.06	0.2034
**CRYGN**	20349.74	12775.33	0.2007
**ρ-crystallin**	26997.95	16695.58	0.1916
**CRYZ**	38.44	22.51	0.1884
